# Association between laser-assisted hatching and subsequent blastocyst development in fresh day 3 cleavage-stage embryos: a retrospective cohort study using propensity score matching, generalized estimating equations, and time-sensitivity analyses

**DOI:** 10.3389/fendo.2026.1871377

**Published:** 2026-07-08

**Authors:** Lixia Miao, Lian Zou, Min Wang, Meiling Weng, Yun Zhang, Xin Jin, Fang Xiong, Huiming Zeng

**Affiliations:** Wuxi Maternity and Child Health Care Hospital, Affiliated Women’s Hospital of Jiangnan University, Wuxi, Jiangsu, China

**Keywords:** blastocyst formation rate, cleavage stage embryo on D3, GEE, LaH, PSM, time-sensitivity analysis

## Abstract

**Background:**

To investigate the association between laser-assisted hatching (LAH) and blastocyst developmental outcomes of day 3 (D3) cleavage-stage embryos in fresh IVF cycles, using Propensity Score Matching (PSM), Generalized Estimating Equation (GEE) analysis, and Time-sensitivity analyses.

**Methods:**

The clinical data of 4365 couples who received *in vitro* fertilization (IVF) treatment at Wuxi Maternal and Child Health Hospital from January 2018 to December 2023 were retrospectively analyzed. The patients were divided into the LAH (n=3193) and the non-LAH (n=1172). To control potential confounding factors, the baseline characteristics of the two groups were balanced by 1:1 Propensity Score Matching (PSM), and 999 couples were included in each group. The Generalized Estimating Equation (GEE) was further applied to correct the multi-factor interference and evaluate the independent effect of LAH on blastocyst development. In this study, the research cohort was restricted within ±6-month, ± 12-month, and ±24-month time windows, and time-sensitivity analyses were performed to assess potential time-related confounding bias.

**Results:**

After propensity score matching, LAH was associated with a higher transferable blastocyst formation rate, especially in grade II–IV embryos (P < 0.001). GEE analysis confirmed this association, whereas no significant improvement in high-quality blastocyst formation was observed. Sensitivity analyses using ±6-, ± 12-, and ±24-month windows showed no significant difference in overall blastocyst formation. For transferable blastocyst formation, no association was found in the ±6-month model, while positive associations were observed in the ±12-month model (adjusted rate difference: 4.62%, 95% CI: 1.30%–7.95%, P = 0.006) and ±24-month model (6.69%, 95% CI: 4.46%–8.92%, P < 0.001). However, after adjustment for calendar time, this association became non-significant (P = 0.573), suggesting possible time-related confounding.

**Conclusion:**

LAH may improve transferable blastocyst formation in low-grade day 3 cleavage-stage embryos under specific analytical conditions, but the findings were heterogeneous across sensitivity analyses. Because implantation, pregnancy, live birth, neonatal safety, and long-term offspring outcomes were not assessed, these results should be considered hypothesis-generating and require confirmation in multicenter prospective randomized trials.

## 1 Introduction

LAH is a key micromanipulation technology in Assisted Reproductive Technology (ART). Its core principle is to precisely target the Zona Pellucida (ZP) of embryos using a diode laser (pulse duration 0.2-2.2 ms) with a wavelength of 1.48 μm (1). According to the differences of operation modes, LAH can be divided into four technical paths: Drilling LAH (D-LAH), Thinning LAH (T-LAH), Partial Removal LAH (P-LAH), and Full-thickness removal LAH (F-LAH) (2–4). LAH aims to reduce the mechanical resistance of the ZP to facilitate the natural hatching process, assist embryos in breaking through the ZP to complete implantation, thereby improving embryo implantation potential and the clinical pregnancy rate. As a glycoprotein bilayer structure that wraps oocytes and early embryos, the ZP, with a thickness of about 15 μm (5), plays a vital role in preventing polyspermy during fertilization and protecting embryo development during natural fertilization (6). However, during IVF, embryo culture, freezing, and other procedures may lead to zona pellucida hardening (7), hindering the natural hatching of embryos. This phenomenon is especially significant in patients who are of advanced age, have experienced repeated implantation failure, or are receiving Frozen Embryo Transfer (FET), and it has become a key factor in embryo implantation failure in ART. To solve this problem, Cohen et al. (8) described assisted hatching (AH) as a micromanipulation technique designed to modify the zona pellucida, either by breaching or thinning it, to potentially aid embryo development. With its advantages of accuracy and high safety, it has rapidly become the mainstream auxiliary incubation method in clinical practice.

Although LAH has been widely adopted in assisted reproductive technology (ART), its optimal clinical application strategies remain a subject of ongoing debate. The conflicting data reported in existing literature can be systematically categorized into three core dimensions: target populations, technical modalities, and clinical outcomes. First, regarding the target population, consensus has not yet been fully reached. For instance, a randomized controlled trial (RCT) study by Kanyo et al. (9) indicated that LAH did not significantly improve implantation rates (IR) or clinical pregnancy rates (CPR) in fresh IVF-ET cycles among advanced-age patients; Similarly, a multicenter double-blind randomized controlled trial by Curfs et al. (10) confirmed that for IVF patients with recurrent implantation failure, AH did not significantly improve the cumulative live birth rate. Second, concerning technical modalities, no universal agreement has been established on the superior approach. A meta-analysis by Chen et al. (2) revealed no significant difference in CPR between drilling LAH (D-LAH) and thinning LAH (T-LAH); although D-LAH showed a slightly higher singleton pregnancy rate, current evidence remains insufficient to establish the absolute superiority of one technique over another. Finally, in terms of clinical outcomes, the reported benefits exhibit high heterogeneity by Sciorio et al. (11) highlighted the positive role of LAH in enhancing pregnancy probabilities, and a meta-analysis by Zeng et al. (12) Meta-analysis results showed that, in frozen-thawed embryo transfer cycles, laser-assisted hatching may improve the clinical pregnancy rate and embryo implantation rate, embryo implantation rate and multiple pregnancy rate of women with frozen thawed embryo transfer, but had no significant effect on the live birth rate and abortion rate. Conversely, a large-sample retrospective study (n=4637) by Jiang et al. (13) found that LAH did not significantly improve the overall implantation rate. These inconsistent pieces of evidence are also reflected in the differences in academic guidelines: the 2023 guideline of the European Society of Human Reproduction and Embryology (ESHRE), together with evidence from the Cochrane systematic review, does not recommend the routine use of assisted hatching in all assisted reproduction populations (14, 15), while the ASRM recommends careful application in a specific FET cycle (16).

There is significant heterogeneity in current LAH clinical research: different studies often combine embryos in various states (fresh or frozen, cleavage-stage or blastocyst), different fertilization methods [IVF or ICSI (Intracytoplasmic Sperm Injection)], and populations with diverse clinical features (such as elderly patients or those with repeated implantation failures). Additionally, there is a lack of standardized technical procedures, like how to handle the zona pellucida and which developmental stage of the embryo to target. Importantly, baseline patient characteristics (such as age, BMI, hormone levels, duration of infertility, and ovulation induction protocols) can significantly influence research outcomes. For example, the rate of embryo aneuploidy is notably higher in older women (17), and patients with elevated BMI often have a less stable embryo metabolic microenvironment (18). Although some existing studies have recognized the potential impact of these confounding factors, systematic control through rigorous baseline matching or advanced statistical adjustments in large-sample cohorts remains relatively limited, which may partially account for the heterogeneity observed in current conclusions. Moreover, there is a lack of systematic evidence on how LAH procedures affect blastocyst developmental potential.

In retrospective studies in the field of assisted reproduction, time-related confounding is a common and important source of bias. During a long study period, changes in the culture system, laboratory environment, operator experience, and patient baseline characteristics may all affect embryonic developmental outcomes. Therefore, calendar time was incorporated into the sensitivity analyses in this study to evaluate the stability of the observed associations under different temporal conditions. To address the limitations of previous studies, the present study was based on a large single-center dataset and uniformly included grade I–IV cleavage-stage embryos on day 3 of fresh *in vitro* fertilization cycles. Multivariable statistical models were used to adjust for key confounders, including female age, body mass index, and hormone levels, to systematically evaluate the association of LAH with embryonic developmental outcomes, including blastocyst formation rate (≥3CC), transferable blastocyst formation rate (≥3BC), and high-quality blastocyst formation rate (≥3BB).

In summary, using fresh day 3 cleavage-stage embryos as the study population, this study combined PSM, multivariable GEE models, and time-sensitivity analyses to adjust for baseline differences and time-related confounding bias, aiming to systematically evaluate the association between LAH and blastocyst developmental outcomes.

## 2 Materials and methods

### 2.1 Research object and grouping

This is a retrospective analysis planned by the reproductive medicine center of Wuxi maternal and child health hospital, covering the clinical data of all patients undergoing IVF treatment from January 2018 to December 2023. After strict inclusion criteria and exclusion criteria, we successfully included 4365 infertile couples who received IVF. It is worth noting that from May 2020 to December 2023, the center implemented LAH technology for embryos cultured on the third day of the fresh cycle. Before that, D3 embryos cultured on the third day of the fresh cycle were not subjected to LAH, and these patients were included in the non-LAH control group (1172 pairs). Conversely, after the introduction of LAH, all patients underwent embryo-assisted hatching. These patients were divided into the LAH group (3193 cases). Because embryos in the two groups were derived from different treatment periods, the present study fully considered, during the statistical analysis, the time-related confounding effects caused by temporal changes in laboratory procedures, embryo culture conditions, incubator systems, and embryologist experience. To minimize the potential influence of confounding factors on blastocyst developmental outcomes, a multivariable confounding-adjustment model was constructed to evaluate the independent effect of laser-assisted hatching on blastocyst formation. Key baseline variables, including male age, female age, duration of infertility, male BMI, female BMI, basal FSH, estradiol (E_2_), prolactin (PRL), LH, and ovarian stimulation protocol, were included in the adjusted analyses. In addition, baseline endocrine parameters measured on menstrual cycle days 2–3, including FSH, E_2_, PRL, LH, and AMH, were collected and incorporated into the statistical models. To evaluate the robustness of the results against potential time-related confounding, additional sensitivity analyses were performed using the oocyte retrieval date as the time scale. The implementation date of laser-assisted hatching at our center, May 15, 2020, was defined as the index date. Study cohorts were reconstructed within symmetric time windows of ±6 months, ± 12 months, and ±24 months around this date. Multivariable generalized estimating equation models were then fitted within each time window, with adjustment for the original clinical covariates and calendar time where appropriate, to account for potential secular trends and clustering of embryos within the same patient/cycle.

Embryo culture was performed using commercial PLUS series media from Vitrolife, including G-IVF™ PLUS, G-1™ PLUS, G-2™ PLUS, and G-MOPS™ PLUS. During the study period, culture media batches were routinely changed according to standard laboratory procedures, and each batch was subjected to strict quality control. No major changes were made to the embryo culture system, culture protocol, or laboratory workflow. Laser-assisted hatching was performed using a standardized non-contact infrared diode laser system. The laser wavelength was 1480 nm, and the standard output power was approximately 400 mW. According to zona pellucida characteristics, the pulse duration was set between 0.38 and 0.58 ms. A non-contact opening was created at the region with the widest perivitelline space, producing an opening of approximately 4.0–6.9 μm. All procedures were performed by experienced embryologists according to a unified standard operating procedure. Embryo culture was conducted in incubators managed under the same laboratory quality-control system. During the study period, two EC6S series incubators were replaced. The culture environment was strictly controlled at 37.0 ± 0.1 °C, 6.0% ± 0.2% CO_2_, and 5.0% ± 0.2% O_2_, with continuous environmental monitoring and standardized quality-assurance procedures. Embryo grading was performed according to the 2011 Istanbul consensus criteria throughout the study period. Although one embryologist changed position during the study period, all embryology procedures, personnel training, grading criteria, and quality-control protocols remained standardized. To assess the robustness of the results against potential time-related confounding, additional sensitivity analyses were performed using the oocyte retrieval date as the time scale. The implementation date of LAH, May 15, 2020, was defined as the index date, and the cohorts were reconstructed within symmetric ±6-month, ± 12-month, and ±24-month windows. Multivariable GEE models were fitted within each time window, with adjustment for clinical covariates and calendar month where appropriate.

This study strictly abides by the ethical norms and has been approved by the reproductive ethics committee of Wuxi maternal and child health hospital with the review number of YLSL2025-071.

### 2.2 Grouping criteria

#### 2.2.1 Inclusion criteria

(1) Micro stimulation scheme or antagonist scheme or long scheme (2); Patients receiving IVF assisted pregnancy therapy (3); The patients were 20 years old or older (4); Data integrity: the patient’s clinical data, laboratory test results and relevant treatment records are complete, including hormone levels, follicular monitoring data, embryonic development records, etc (5); A sufficient number of embryos at D3 cleavage stage can be used for subsequent blastocyst culture, that is, cultured to Day 5 (D5)/Day 6 (D6) for blastocyst evaluation, and the quality evaluation data of these embryos are complete.

#### 2.2.2 Exclusion criteria

(1) Patients with ICSI fertilization should avoid the potential interference of sperm injection technology on embryonic development, and focus on the role of LAH in natural fertilization embryos (2); Patients undergoing remedial ICSI fertilization due to IVF fertilization failure (3); Important clinical data or laboratory test results are missing, such as key hormone level data (estradiol, luteinizing hormone, etc.), follicular monitoring results, and incomplete embryo score records (4); There is a gap in the relevant data required for the study, which makes it impossible to conduct a complete data analysis (5); Patients with no blastocyst culture strategy or no extra cleavage embryos available for blastocyst culture (6); Severe ovarian dysfunction: severe ovarian dysfunction, such as a significant increase in basal FSH and a very low level of AMH, resulting in a small number of embryos (7); Genetic factors: there are known genetic diseases or chromosome abnormalities that may affect the quality of embryos and pregnancy outcomes.

### 2.3 Oocyte retrieval, IVF, and embryo culture

According to ESHRE, 2023, combined with age, ovarian reserve (such as AMH, antral follicle count (AFC)), etiology and history of infertility, individualized schemes such as gonadotropin-releasing hormone (GnRH) agonist long scheme, antagonist scheme or micro stimulation scheme were selected to regulate pituitary function: GnRH agonist long scheme was suitable for patients with normal ovarian response, and the recombinant FSH was activated after down regulation; antagonists can reduce the risk of premature LH peak in patients with polycystic ovary syndrome; for those with low ovarian reserve, low-dose gonadotropin combined with oral drugs was used to induce follicular development. During ovulation induction, the levels of serum E_2_, LH, and the diameter of follicles under transvaginal ultrasound were dynamically monitored. When ≥2 follicles reached 16mm, the follicles were mature. At this time, the doctor gave the patient an appropriate dose of human chorionic gonadotropin (hCG) to trigger (or induce) final follicular maturation and ovulation. At 36 hours after hCG injection, i.e., 36 hours after trigger, the oocytes were retrieved under the guidance of transvaginal ultrasound under intravenous anesthesia. Follicles ≥14 mm were aspirated with a 17-gauge puncture needle. The extracted follicular fluid will be quickly transferred to the embryo laboratory. The embryologist quickly retrieved the oocyte-corona-cumulus complex (OCCC) under a stereomicroscope (10 ×) at 37 °C and moved it to a Petri dish containing G-IVF™ PLUS medium for temporary storage in a constant-temperature incubator at 37 °C, 6% CO_2_, 5% O_2_, and 89% N_2_. 2–4 hours after oocyte retrieval, the doctor chooses the fertilization method based on sperm optimization results (such as density and forward movement rate) and the couple’s medical history: conventional IVF or ICSI to complete fertilization. At 4–6 hours after fertilization, the IVF group embryos were stripped of granulosa cells, and the unbound sperm and peripheral granulosa cells were mechanically removed for subsequent observation.

After fertilization, the day of oocyte retrieval was designated Day 0 (D0). The embryonic development was evaluated day by day: Day 1 (D1) observed the pronuclear morphology to judge regular fertilization, Day 2 (D2)- D3 scored the morphology according to the number of blastomeres, fragmentation rate and cell uniformity, D5 - D6 combined with Gardner grading standard to evaluate the quality of blastocyst development stage (≥ three phases), inner cell mass (ICM) and trophectoderm (TE), and finally screened high-quality embryos for transfer or freezing through the scoring system of dynamic development dynamics and static morphology, so as to lay the foundation for subsequent pregnancy.

### 2.4 Embryo scoring criteria

#### 2.4.1 Scoring criteria for cleavage embryos

1. Grade I embryos: the number of cells is 7–10 blastomeres, which is in line with the ideal development rate on the third day after fertilization; the size of the blastomere is uniform, the cytoplasm is uniform and transparent, and the fragmentation rate is ≤ 5%.
2. Grade II embryos: the number of blastomeres is 6-10, but the size is uniform; or the fragment rate is between 5% and 10%; or the size of the blastomere was slightly uneven, but there was no significant morphological abnormality.
3. Grade III embryo: the size of the blastomere is uneven, and the shape is irregular, and the fragment rate is less than 10%; or the fragment rate is 10% -20%; or the number of cells is still 4-5, and the size is uniform without debris. Embryos of this grade need to be comprehensively evaluated in combination with developmental dynamics (such as division speed).
4. Grade IV embryo (low-quality embryo): the size of the blastomere is seriously uneven, and the fragmentation rate is more than 20%.

#### 2.4.2 Routine evaluation criteria for blastocyst stage embryos

Blastocyst-stage embryos are routinely evaluated using a standardized six-stage morphological system (stages 1–6) that reflects blastocoel expansion and hatching status. Stage 1 corresponds to the early blastocyst, characterized by a blastocoel that occupies less than half of the embryo’s volume. Stage 2 is identified by a blastocoel that exceeds 50% of the embryo’s volume but is not yet fully expanded. Stage 3 shows complete blastocoel expansion, with the cavity filling the entire embryo and the zona pellucida remaining intact. In Stage 4, continued expansion results in increased overall embryo size and thinning of the zona pellucida. Stage 5 shows active hatching, with TE cells protruding through the zona pellucida. Stage 6 describes a fully hatched blastocyst that has entirely emerged from the zona pellucida.

In addition to developmental staging, morphological evaluation includes grading of the ICM and TE. The ICM is graded as Grade A when it consists of a tightly packed, well-defined cluster of more than 30 cells; Grade B indicates a moderately sized ICM (10–30 cells) with looser cellular organization and some gaps; and Grade C denotes a small, poorly defined ICM with fewer than 10 cells. Similarly, TE quality is graded based on cell density and cohesion: Grade A contains more than 50 tightly packed cells forming a uniform epithelium; Grade B comprises 20–50 cells with moderate continuity; and Grade C is assigned when fewer than 20 cells form a discontinuous or sparse layer. This comprehensive grading system enables a more precise evaluation of blastocyst quality and developmental potential.

### 2.5 Culture system pretreatment

#### 2.5.1 Medium pretreatment

On the sterile operating table, place a 35-mm petri dish and prepare 8 50 μL droplets of G-II-Plus blastocyst medium. Then cover the surface with 3 mL of mineral oil to stabilize the droplets and prevent contamination. Place the Petri dish in a constant-temperature incubator at 37°C and 6% CO_2_ for at least 12 h to ensure optimal medium temperature, pH, and osmotic pressure for embryonic development.

#### 2.5.2 Embryo evaluation and co-culture grouping

When the embryo reached D3, its quality was graded under an inverted microscope based on cell count, fragmentation rate, and cell-sphere uniformity. After full communication with patients, patients’ embryos that met the indications for blastocyst culture were screened and grouped by quality level using the cluster co-culture mode; that is, 3–5 embryos of the same level were transferred into pretreated medium droplets. The best position for LAH is determined by experienced embryologists: the target site is usually selected as the weak structural area of the ZP or the position with a large Perivitelline Space (PVS), to ensure the normal developmental potential of embryos and minimize the risk of cell damage from laser energy.

### 2.6 Equipment calibration and parameter setting

Before using the special LAH system (ZILOS-tk^®^, Hamilton Thorne, USA; a non-contact diode laser system), it is necessary to calibrate the wavelength, power, and focus accuracy of the laser emission system: set the ablation laser parameters to 1480 nm and 400 mW. The non-contact laser beam is delivered through the microscope objective lens without physically contacting the embryo or culture dish, thereby minimizing the risk of contamination and mechanical trauma. According to the ZP thickness (5-15 μm) and PVS width, dynamically adjust the operation parameters: the laser exposure time is set between 0.38 and 0.58 milliseconds (typically requiring 2–5 sequential laser pulses per embryo to create a single circular aperture) to ensure that the aperture formed is within the range of 4.0 to 6.9 μm, and the aperture diameter cannot exceed the thickness of the transparent belt zona pellucida itself. The drilling location was selected at the site with the widest perivitelline space (PVS), avoiding the inner cell mass (ICM) region and areas with cellular debris or fragmentation, as identified by an experienced embryologist under 400× magnification. To minimize thermal damage, the cutting site should be selected at the broadest part of the periovum space, and, at the same time, the energy-delivery path should avoid the ICM core cell mass of the embryo. After the operation, the Petri dish must be immediately returned to the 37 °C incubator containing 5% oxygen and 6% carbon dioxide, and the *in vitro* exposure time of embryos must be strictly controlled, not to exceed 5 min. Through mineral oil coverage and precise gas environment control, the microenvironment of the culture medium was maintained stable, minimizing the influence of external interference on blastocyst formation. All LAH procedures were performed by two senior embryologists (each with >3 years of micromanipulation experience).

### 2.7 Calculation formula

Blastocyst formation rate: number of developed blastocysts/number of cultured blastocysts * 100%.

Transferable blastocyst rate: number of transferable blastocysts/number of cultured blastocysts * 100%.

High-quality blastocyst rate: number of high-quality blastocysts/number of cultured blastocysts * 100%.

### 2.8 Statistical methods

**Figure 1:**
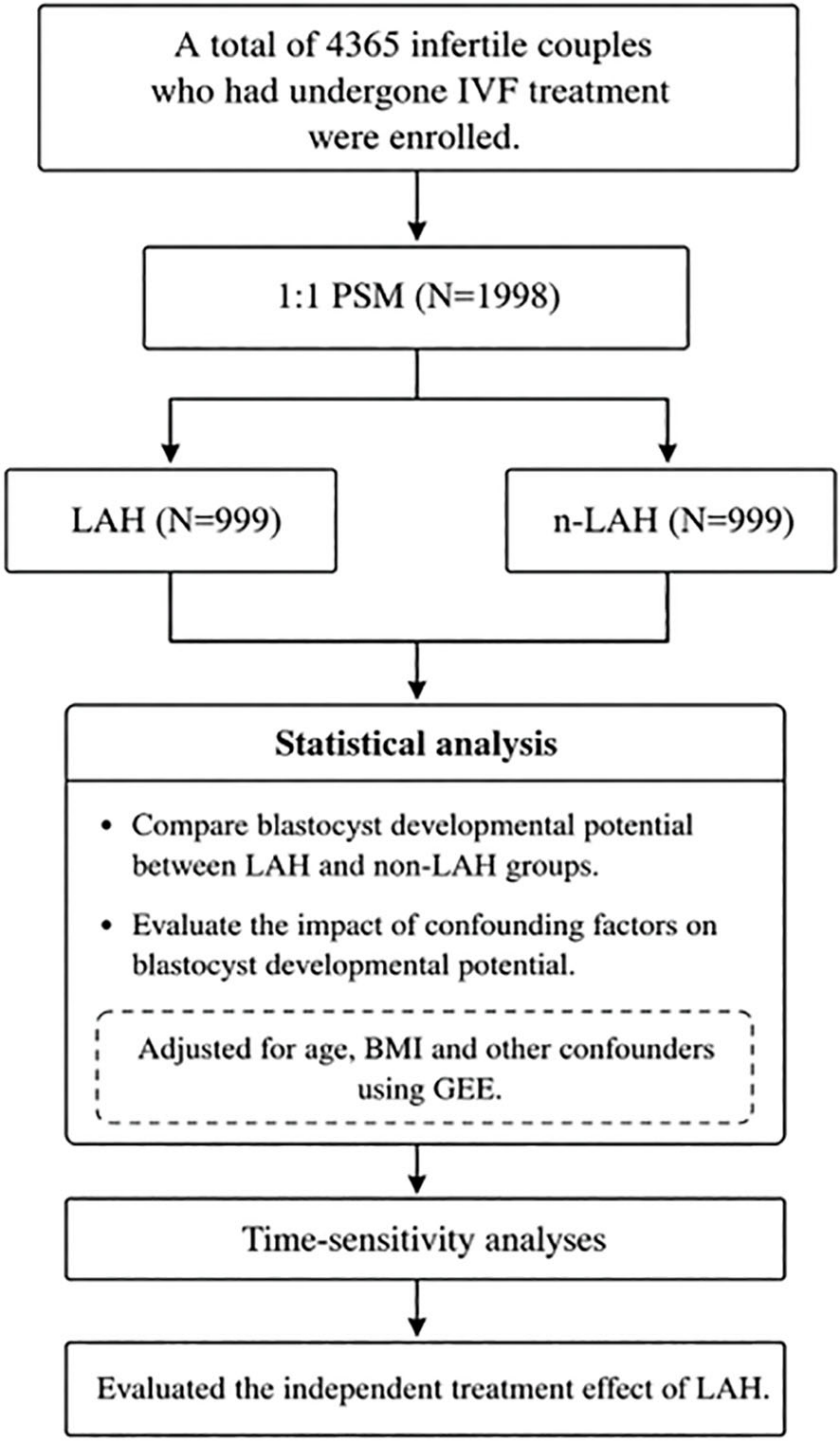
Flowchart of study design. This study included 4,365 couples undergoing IVF treatment. After 1:1 propensity score matching, 999 cases were assigned to LAH group and 999 to the non-LAH group.

Based on predefined inclusion and exclusion criteria, a total of 4,365 infertile couples undergoing IVF treatment were retrospectively enrolled as the initial study cohort. To minimize selection bias and ensure comparability of baseline characteristics between groups, PSM was performed via a multivariable binary logistic regression model using a nearest-neighbor algorithm with a 1:1 matching ratio and a caliper width of 0.2 of the standard deviation of the logit of the propensity score. After matching, 1,998 cycles were successfully included, comprising 999 cases in the LAH group and 999 cases in the non-LAH group, as illustrated in the study flowchart (**Figure 1**).

PSM incorporated key baseline covariates including female age, male age, female BMI, male BMI, infertility type, treatment protocol, duration of infertility, and baseline endocrine parameters, including FSH, E_2_, PRL, LH, and AMH. Balance between groups was assessed using standardized mean differences (SMDs), with values <10% indicating adequate matching, supplemented by traditional hypothesis testing to confirm the successful elimination of statistically significant intergroup differences (P > 0.05).

After testing for normality using the Shapiro-Wilk test, continuous variables and patient-level blastocyst developmental outcome proportions were summarized as medians with interquartile ranges [median (IQR)], because normality testing indicated non-normal distributions. In addition, blastocyst developmental outcomes were proportions bounded between 0 and 1, with skewed distributions in several outcomes. Continuous variables were compared using the Mann-Whitney U test, while the Kruskal-Wallis H test was applied for comparisons among multiple groups. Categorical variables were expressed as counts and percentages [n (%)] and compared using the chi-square test or Fisher’s exact test when expected cell counts were <5. Spearman’s rank correlation analysis was performed to evaluate associations between continuous clinical variables and blastocyst development parameters.

To account for the clustering effect of multiple sister embryos derived from the same couple, GEE models were subsequently applied to assess the independent effect of LAH on blastocyst formation rate, transferable blastocyst rate, and high-quality blastocyst rate, with adjustment for potential confounders including female age, male age, BMI, infertility type, treatment protocol, infertility duration, and baseline hormone levels (FSH, E_2_, PRL, LH, and AMH). An independent working correlation matrix with robust variance estimation was used to account for potential intra-group correlation. The adjusted differences and 95% confidence intervals (95% CIs) were calculated. To effectively control for potential time-related confounding bias, the oocyte retrieval time variable was incorporated into the multivariable models for sensitivity analyses. Through calendar-month adjustment and fixed-effect analysis of the oocyte retrieval year, the robustness of the association between LAH and blastocyst developmental outcomes was further evaluated after adjusting for the potential influence of temporal changes in laboratory operation levels. Statistical analyses were performed using Python 3.11, SPSS 26.0, and R 4.3.2, and figures were generated using GraphPad Prism 9.0. All statistical tests were two-sided, and *P* < 0.05 was considered statistically significant.

## 3 Results

### 3.1 Schematic diagram of the human embryonic development process and LAH operation

**Figure 2:**
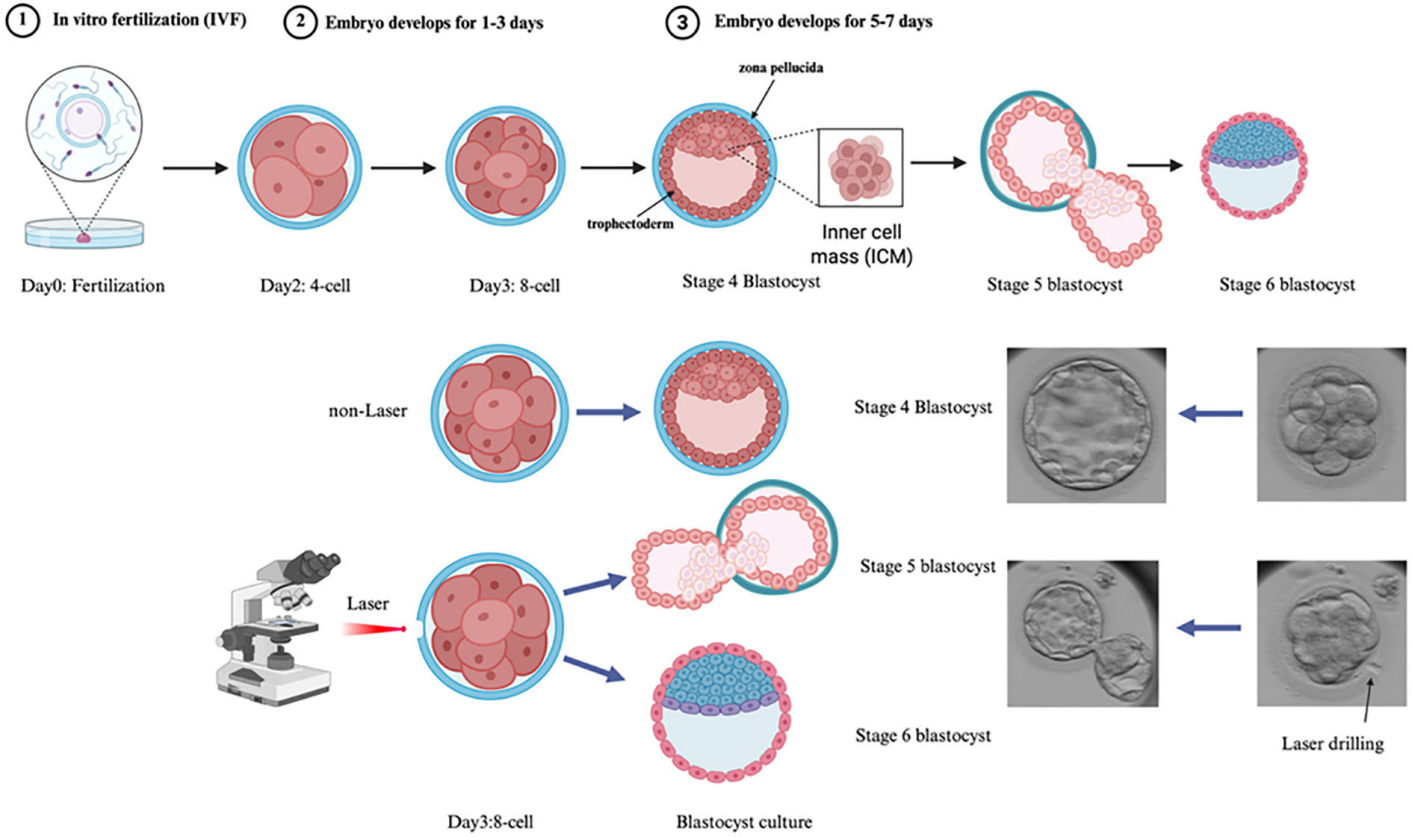
Schematic diagram of human embryo development and LAH procedure.

As shown in the **Figure 2**, after IVF, embryos develop to the 4-cell and 8-cell stages on D2 and D3, respectively. In the subsequent blastocyst culture stage (day 5-7), the embryo underwent stage 4 (expanded blastocyst, not hatched), stage 5 (hatching blastocyst), and stage 6 (fully hatched blastocyst). This study evaluated the effect of LAH treatment on the development of D3 embryos into blastocysts. The results showed that blastocysts formed by embryos that had not undergone LAH treatment were mainly distributed at stage 4 when cultured for the day 5; after LAH treatment, most blastocysts developed to stage 5 or 6. The results showed that LAH treatment promoted blastocyst hatching.

### 3.2 Comparison of baseline characteristics of subjects’ propensity score before and after matching

Before PSM, there were statistically significant differences (all *P* < 0.05) between the LAH group and the non-LAH group in key baseline variables such as male age, female age, years of infertility, male BMI, female BMI, basal FSH, E_2_, PRL, LH and the type of ovulation induction program, suggesting that the initial sample had the risk of selection bias (**Table 1**). In addition, relatively larger SMD values were observed for female age, FSH, E_2_, and treatment approach before matching, indicating baseline imbalance between groups. After 1:1 nearest-neighbor matching, 999 cycles were retained in each group, and there was no significant difference between the two groups (all *P* > 0.05). Furthermore, all post-matching SMD values were <0.10, indicating satisfactory covariate balance after matching.

**Table 1:** Comparison of baseline characteristics before and after propensity score matching of the research subjects.

Index	Before PSM					After PSM				
	N-LAH n=1172	LAH n=3193	SMD	*Z/χ²*	*P*	N-LAH n=999	LAH n=999	SMD	*Z/χ²*	*P*
Female partner's age (years)				-4.544	<0.001				-0.504	0.614
<35	969(82.7%)	2441(76.4%)	0.155			818(81.9%)	810(81.1%)	0.021		
35-39	163(13.9%)	563(17.6%)	0.102			144(14.4%)	146(14.6%)	0.006		
>39	40(3.4%)	189(5.9%)	0.119			37(3.7%)	43(4.3%)	0.031		
Male partner's age (years)				-2.763	0.006				-0.904	0.366
<35	871(74.3%)	2243(70.2%)	0.091			735(73.6%)	716(71.7%)	0.043		
35-39	212(18.1%)	638(20%)	0.048			181(18.1%)	197(19.7%)	0.041		
>39	89(7.6%)	312(9.8%)	0.077			83(8.3%)	86(8.6%)	0.011		
Infertility duration (years)	3.25(2,5)	3.08(1.83,4.92)	0.044	-1.987	0.047	3.33(2,5)	3.17(1.92,5)	0.024	-0.931	0.352
Female partner's BMI(kg/m²)				-0.688	0.491				-0.595	0.552
18.5-24.9	958(81.7%)	2580(80.8%)	0.024			814(81.5%)	824(82.5%)	0.026		
25.0-29.9	198(16.9%)	570(17.9%)	0.025			171(17.1%)	163(16.3%)	0.021		
≥30	16(1.4%)	43(1.3%)	0.002			14(1.4%)	12(1.2%)	0.018		
Male partner's BMI(kg/m²)				-3.076	0.002				-0.424	0.672
18.5-24.9	699(59.6%)	1762(55.2%)	0.090			582(58.3%)	596(59.7%)	0.028		
25.0-29.9	416(35.5%)	1190(37.3%)	0.037			363(36.3%)	340(34%)	0.048		
≥30	57(4.9%)	241(7.5%)	0.111			54(5.4%)	63(6.3%)	0.038		
FSH	6.7(5.6,8.05)	7.65(6.51,9.1)	0.421	-14.542	<0.001	6.87(5.69,8.2)	6.98(6,8.22)	0.011	-1.635	0.102
E2	42.08(33,53)	29.3(22,39.1)	0.543	-22.783	<0.001	40(32,49.4)	37.9(27.7,52.9)	0.049	-1.822	0.068
PRL	13.81(10.14,18.34)	12.95(9.69,17.47)	0.074	-3.175	0.001	13.89(10.18,18.25)	13.21(9.96,18.06)	0.012	-1.075	0.283
LH	4.53(3.34,6.35)	4.3(3.08,6.06)	0.062	-3.511	<0.001	4.52(3.37,6.32)	4.34(3.16,6.18)	0.047	-1.842	0.066
AMH	3.59(1.99,5.95)	3.8(2.11,6.23)	0.050	-1.23	0.219	3.59(1.91,6.03)	3.7(2.23,5.82)	0.028	-0.748	0.455
Infertility types				0.254	0.614				0.05	0.823
Secondary infertility	575(49.1%)	1594(49.9%)	0.017			490(49%)	495(49.5%)	0.010		
Primary infertility	597(50.9%)	1599(50.1%)	0.017			509(51%)	504(50.5%)	0.010		
Treatment approach				24.431	<0.001				0.583	0.445
Stimulation cycle	881(75.2%)	2152(67.4%)	0.172			730(73.1%)	745(74.6%)	0.034		
Mild stimulation cycle	291(24.8%)	1041(32.6%)	0.172			269(26.9%)	254(25.4%)	0.034		

LAH, laser-assisted hatching; BMI, body mass index; FSH, follicle-stimulating hormone; E_2_, estradiol; PRL, prolactin; LH, luteinizing hormone; AMH, anti-Müllerian hormone; SMD, standardized mean difference; IQR, interquartile range.

### 3.3 Associations between clinical characteristics, LAH treatment, and blastocyst developmental outcomes

**Figure 3:**
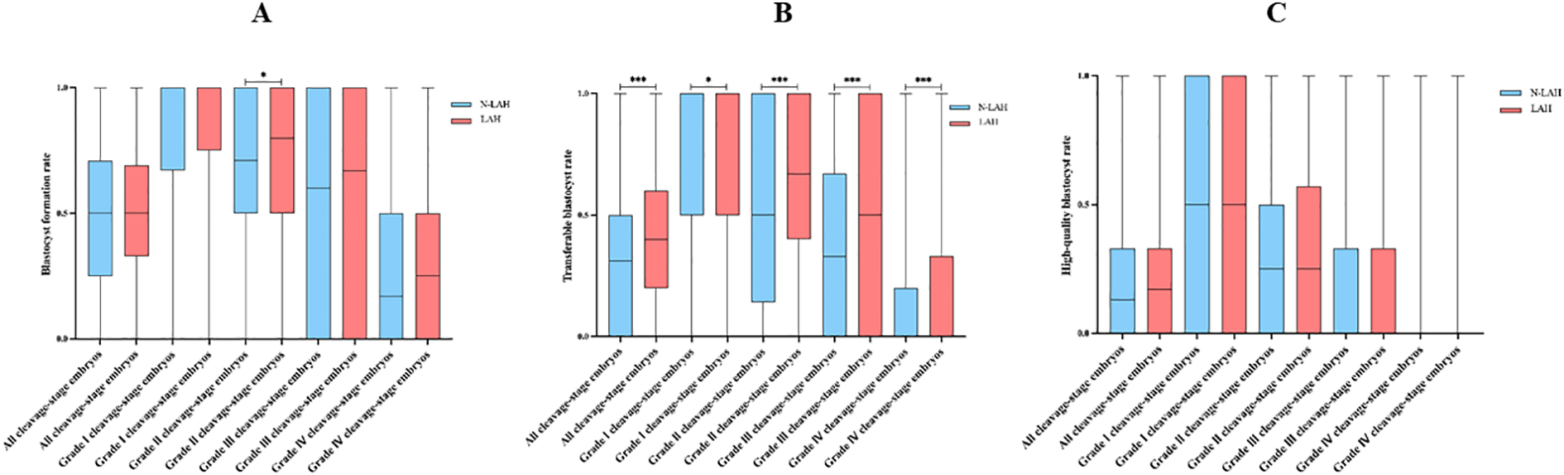
Distribution and intergroup differences in blastocyst outcome measures between the LAH and non-LAH Groups. **(A)** Overall blastocyst formation rate; **(B)** Transferable blastocyst formation rate; **(C)** High-quality blastocyst formation rate.

The analysis of key influencing factors in blastocyst development is shown in **Table 2**, **Supplementary Tables 1**–**6**, and **Figure 3**.

**Table 2:** Comparison of blastocyst development outcomes between LAH and non-LAH groups.

Embryo grade & outcome measure	Non-LAH (n = 999) Median (IQR), 95% CI	LAH (n = 999) Median (IQR), 95% CI	Z	|r| (95% CI)	*P* value	Adjusted P value†
Blastocyst formation, all stages	0.5 (0.25, 0.714); 95% CI: 0.5–0.5	0.5 (0.333, 0.692); 95% CI: 0.5–0.5	-1.926	0.043 (-0.087, 0.001)	0.054	0.116
Transferable blastocyst, all stages	0.308 (0, 0.5); 95% CI: 0.273–0.333	0.4 (0.2, 0.6); 95% CI: 0.375–0.433	-7.316	0.164 (-0.206, -0.121)	<0.001	<0.001
High-quality blastocyst, all stages	0.133 (0, 0.333) 95% CI: 0.1–0.167	0.167 (0, 0.333); 95% CI: 0.143–0.176	-1.323	0.03 (-0.073, 0.014)	0.186	0.310
Blastocyst formation, Grade I	1 (0.667, 1) 95% CI: 1–1	1 (0.75, 1); 95% CI: 1–1	-1.428	0.064 (-0.151, 0.024)	0.154	0.288
Transferable blastocyst, Grade I	1 (0.5, 1); 95% CI: 0.678–1	1 (0.5, 1); 95% CI: 1–1	-2.2	0.098 (-0.185, -0.011)	0.028	0.081
High-quality blastocyst, Grade I	0.5 (0, 1) 95% CI: 0.5–0.5	0.5 (0, 1); 95% CI: 0.5–0.6	-0.124	0.006 (-0.093, 0.082)	0.901	0.901
Blastocyst formation, Grade II	0.714 (0.5, 1); 95% CI: 0.667–0.75	0.8 (0.5, 1); 95% CI: 0.75–0.833	-2.141	0.058 (-0.111, -0.005)	0.032	0.081
Transferable blastocyst, Grade II	0.5 (0.143, 1); 95% CI: 0.5–0.5	0.667 (0.4, 1); 95% CI: 0.625–0.667	-5.513	0.150 (-0.202, -0.098)	<0.001	<0.001
High-quality blastocyst, Grade II	0.25 (0, 0.5); 95% CI: 0.2–0.333	0.25 (0, 0.571); 95% CI: 0.25–0.333	-1.199	0.033 (-0.086, 0.021)	0.231	0.314
Blastocyst formation, Grade III	0.6 (0, 1); 95% CI: 0.5–0.667	0.667 (0, 1); 95% CI: 0.5–0.667	-0.799	0.023 (-0.079, 0.034)	0.425	0.531
Transferable blastocyst, Grade III	0.333 (0, 0.667); 95% CI: 0–0.333	0.5 (0, 1); 95% CI: 0.481–0.5	-5.115	0.147 (-0.202, -0.092)	<0.001	<0.001
High-quality blastocyst, Grade III	0 (0, 0.333); 95% CI: 0–0	0 (0, 0.333); 95% CI: 0–0	-0.26	0.007 (-0.064, 0.049)	0.795	0.867
Blastocyst formation, Grade IV	0.167 (0, 0.5); 95% CI: 0.097–0.216	0.25 (0, 0.5); 95% CI: 0.174–0.25	-1.221	0.031 (-0.08, 0.019)	0.222	0.314
Transferable blastocyst, Grade IV	0 (0, 0.2); 95% CI: 0–0	0 (0, 0.333); 95% CI: 0–0	-5.238	0.132 (-0.18, -0.083)	<0.001	<0.001
High-quality blastocyst, Grade IV	0 (0, 0); 95% CI: 0–0	0 (0, 0); 95% CI: 0–0	-0.241	0.006 (-0.055, 0.043)	0.810	0.867

Data are presented as median (interquartile range, IQR) of patient-level proportions. 95% CI: 95% confidence interval of the proportion.

†P values adjusted using the Benjamini-Hochberg false discovery rate (FDR) procedure within each variable.

LAH, laser-assisted hatching; IQR, interquartile range; CI, confidence interval.

As shown in **Table 2**, the LAH group demonstrated significantly higher transferable blastocyst formation rates for all cleavage-stage embryos and for Grade II–IV embryos compared with the non-LAH group (all FDR-adjusted P < 0.001). However, no significant differences were observed in overall blastocyst formation rate or high-quality blastocyst formation rate after FDR correction.

Subgroup analyses are presented in **Supplementary Tables 1**–**6**. As shown in **Supplementary Table 1**, increasing female age was significantly associated with lower overall transferable blastocyst formation rate and high-quality blastocyst formation rate after FDR correction (both adjusted P < 0.001). Significant differences were also observed in several embryo-grade-specific developmental outcomes, particularly high-quality blastocyst formation rates in Grade I–III embryos.

As shown in **Supplementary Table 2**, male age was significantly associated with overall high-quality blastocyst formation rate and several developmental indicators in Grade I, Grade II, and Grade IV embryos after FDR adjustment, whereas most other outcomes were comparable among groups.

As shown in **Supplementary Tables 3**, **4**, female BMI and male BMI were not significantly associated with most blastocyst developmental outcomes after FDR correction.

As shown in **Supplementary Table 5**, most developmental outcomes were comparable between primary and secondary infertility groups after FDR correction, although the high-quality blastocyst formation rate of Grade III embryos remained significantly different between groups (adjusted P = 0.014).

As shown in **Supplementary Table 6**, significant differences were observed between stimulation cycles and mild stimulation cycles in overall high-quality blastocyst formation rate, as well as blastocyst formation, transferable blastocyst formation, and high-quality blastocyst formation rates of Grade IV embryos after FDR correction.

### 3.4 Correlation analysis between continuous clinical variables and blastocyst developmental outcomes

As shown in **Table 3**, infertility duration was negatively correlated with total blastocyst formation rate, total transferable blastocyst formation rate, and total high-quality blastocyst formation rate before FDR correction (RS = -0.048, P = 0.032; RS = -0.061, P = 0.006; and RS = -0.061, P = 0.006, respectively). In addition, infertility duration showed negative correlations with the transferable blastocyst formation rate and high-quality blastocyst formation rate of Grade II cleavage-stage embryos before FDR correction (RS = -0.054, P = 0.048; and RS = -0.061, P = 0.024, respectively). However, these associations were not statistically significant after FDR adjustment.

**Table 3:** Spearman correlation analysis between continuous clinical variables and blastocyst developmental outcomes.

Index		Infertility years (years)	Basic FSH (mIU/mL)	Basic E2(pg/mL)	Basic PRL(ng/mL)	Basic LH (mIU/mL)	AMH(ng/mL)
Total blastocyst formation rate	RS	-0.048	0.003	0.026	-0.027	0.055	0.042
	95% CI	-0.090 to -0.004	-0.049 to 0.048	-0.015 to 0.066	-0.068 to 0.012	0.015 to 0.097	-0.003 to 0.090
	P	0.032*	0.893	0.244	0.228	0.014*	0.062
	FDR-P	0.208	0.971	0.678	0.659	0.149	0.335
Total transferable blastocyst formation rate	RS	-0.061	0.017	0.001	-0.010	0.052	0.045
	95% CI	-0.104 to -0.022	-0.026 to 0.052	-0.043 to 0.051	-0.050 to 0.029	0.010 to 0.096	-0.003 to 0.088
	P	0.006**	0.453	0.993	0.664	0.019*	0.047*
	FDR-P	0.092	0.774	0.993	0.804	0.161	0.291
Total high-quality blastocyst formation rate	RS	-0.061	-0.029	0.015	0.015	0.090***	0.114***
	95% CI	-0.100 to -0.010	-0.072 to 0.023	-0.030 to 0.059	-0.027 to 0.061	0.046 to 0.130	0.075 to 0.159
	P	0.006**	0.188	0.500	0.513	<0.001**	<0.001**
	FDR-P	0.092	0.659	0.774	0.774	0.002††	0.000†††
Blastocyst formation rate of grade I cleavage-stage embryos	RS	0.028	0.121	0.040	-0.032	0.023	-0.011
	95% CI	-0.044 to 0.103	0.046 to 0.198	-0.044 to 0.121	-0.107 to 0.049	-0.063 to 0.103	-0.091 to 0.073
	P	0.536	0.007**	0.376	0.468	0.602	0.814
	FDR-P	0.791	0.107	0.774	0.774	0.804	0.925
Transferable blastocyst formation rate of grade I cleavage-stage embryos	RS	-0.007	0.116	0.054	0.039	0.014	-0.017
	95% CI	-0.081 to 0.080	0.033 to 0.206	-0.032 to 0.138	-0.042 to 0.129	-0.081 to 0.091	-0.096 to 0.073
	P	0.881	0.009**	0.229	0.382	0.763	0.709
	FDR-P	0.991	0.118	0.659	0.774	0.880	0.844
High-quality blastocyst formation rate of grade I cleavage-stage embryos	RS	-0.002	0.028	0.003	0.031	0.022	0.055
	95% CI	-0.082 to 0.081	-0.061 to 0.122	-0.084 to 0.103	-0.062 to 0.113	-0.067 to 0.094	-0.020 to 0.160
	P	0.966	0.525	0.947	0.495	0.618	0.217
	FDR-P	0.993	0.774	0.986	0.774	0.804	0.659
Blastocyst formation rate of grade II cleavage-stage embryos	RS	-0.040	0.045	0.037	0.034	0.016	-0.018
	95% CI	-0.087 to 0.005	-0.002 to 0.098	-0.006 to 0.086	-0.021 to 0.083	-0.035 to 0.075	-0.067 to 0.028
	P	0.143	0.099	0.176	0.216	0.561	0.500
	FDR-P	0.612	0.497	0.659	0.659	0.794	0.774
Transferable blastocyst formation rate of grade II cleavage-stage embryos	RS	-0.054	0.035	0.014	0.014	0.018	0.002
	95% CI	-0.101 to 0.009	-0.017 to 0.082	-0.033 to 0.054	-0.034 to 0.065	-0.041 to 0.068	-0.056 to 0.055
	P	0.048*	0.197	0.609	0.612	0.514	0.936
	FDR-P	0.255	0.659	0.804	0.804	0.774	0.986
High-quality blastocyst formation rate of grade II cleavage-stage embryos	RS	-0.061	0.001	0.030	0.020	0.029	0.024
	95% CI	-0.117 to -0.012	-0.050 to 0.059	-0.019 to 0.082	-0.033 to 0.074	-0.025 to 0.087	-0.028 to 0.078
	P	0.024*	0.978	0.267	0.461	0.280	0.386
	FDR-P	0.181	0.991	0.714	0.774	0.724	0.774
Blastocyst formation rate of grade II cleavage-stage embryos	RS	-0.001	0.036	-0.001	-0.061	0.027	0.013
	95% CI	-0.051 to 0.051	-0.013 to 0.089	-0.052 to 0.047	-0.117 to -0.009	-0.023 to 0.088	-0.046 to 0.065
	P	0.986	0.210	0.964	0.035*	0.358	0.664
	FDR-P	0.993	0.659	0.991	0.237	0.774	0.804
Transferable blastocyst formation rate of grade II cleavage-stage embryos	RS	0.001	0.024	-0.030	-0.040	0.010	0.031
	95% CI	-0.046 to 0.047	-0.027 to 0.081	-0.084 to 0.031	-0.103 to 0.017	-0.046 to 0.066	-0.023 to 0.085
	P	0.970	0.410	0.299	0.168	0.736	0.290
	FDR-P	0.993	0.774	0.724	0.659	0.863	0.724
High-quality blastocyst formation rate of grade II cleavage-stage embryos	RS	-0.037	-0.014	-0.003	-0.017	0.028	0.040
	95% CI	-0.095 to 0.008	-0.067 to 0.042	-0.059 to 0.054	-0.064 to 0.045	-0.027 to 0.079	-0.014 to 0.097
	P	0.197	0.631	0.919	0.551	0.337	0.161
	FDR-P	0.623	0.804	0.985	0.794	0.774	0.659
Blastocyst formation rate of grade IV cleavage-stage embryos	RS	-0.020	-0.048	0.036	-0.005	0.011	0.068
	95% CI	-0.076 to 0.029	-0.094 to 0.000	-0.013 to 0.086	-0.046 to 0.043	-0.035 to 0.054	0.009 to 0.112
	P	0.417	0.057	0.150	0.838	0.661	0.007**
	FDR-P	0.790	0.329	0.659	0.935	0.804	0.107
Transferable blastocyst formation rate of grade IV cleavage-stage embryos	RS	-0.008	-0.023	0.012	-0.018	-0.016	0.055
	95% CI	-0.054 to 0.033	-0.074 to 0.033	-0.040 to 0.066	-0.067 to 0.032	-0.062 to 0.034	0.007 to 0.101
	P	0.746	0.363	0.636	0.475	0.522	0.028*
	FDR-P	0.895	0.774	0.804	0.774	0.774	0.212
High-quality blastocyst formation rate of grade IV cleavage-stage embryos	RS	0.026	-0.059	0.019	0.005	0.016	0.087*
	95% CI	-0.027 to 0.072	-0.100 to -0.006	-0.022 to 0.060	-0.046 to 0.053	-0.031 to 0.071	0.042 to 0.137
	P	0.301	0.019*	0.439	0.848	0.527	0.001**
	FDR-P	0.694	0.161	0.774	0.935	0.774	0.014†

"*" indicates P < 0.05; "**" indicates P < 0.01; "***" indicates P < 0.001; "†" indicates FDR-P < 0.05; "††" indicates FDR-P < 0.01; "†††" indicates FDR-P < 0.001.

RS, Spearman's rank correlation coefficient; CI, confidence interval; FDR-P, false discovery rate-adjusted P value using the Benjamini–Hochberg procedure; FSH, follicle-stimulating hormone; E2, estradiol; PRL, prolactin; LH, luteinizing hormone; AMH, anti-Müllerian hormone.

Statistical significance after multiple-testing correction was defined as FDR-P < 0.05. Associations with nominal significance (P < 0.05) but not surviving FDR correction should be interpreted cautiously.

FSH was positively correlated with the blastocyst formation rate and transferable blastocyst formation rate of Grade I cleavage-stage embryos before FDR correction (RS = 0.121, P = 0.007; and RS = 0.116, P = 0.009, respectively), whereas a negative correlation was observed between FSH and the high-quality blastocyst formation rate of Grade IV embryos (RS = -0.059, P = 0.019). No significant correlations were observed between basal E_2_ levels and blastocyst developmental outcomes. PRL was negatively correlated with the blastocyst formation rate of Grade III embryos before FDR correction (RS = -0.061, P = 0.035). However, these associations did not remain statistically significant after multiple-comparison adjustment.

Among baseline endocrine parameters, LH was positively correlated with total high-quality blastocyst formation rate after FDR correction (RS = 0.090, FDR-adjusted P = 0.002). AMH was also positively correlated with total high-quality blastocyst formation rate (RS = 0.114, FDR-adjusted P < 0.001). In addition, AMH showed a significant positive correlation with the high-quality blastocyst formation rate of Grade IV cleavage-stage embryos after FDR correction (RS = 0.087, FDR-adjusted P = 0.014). Most other embryo-grade-specific developmental indicators were not significantly correlated with infertility duration or endocrine parameters after FDR correction.

### 3.5 Analysis of the independent effect of LAH on the related indexes of blastocyst formation based on the generalized estimation equation

After the baseline variables (including age of male and female, BMI, infertility type, treatment method, infertility years, FSH, E_2_, PRL, LH, AMH, etc.) were corrected by GEE model, the analysis results of the impact of LAH measures on blastocyst rate showed that (**Table 4**): the formation rate of transferable blastocysts of all embryos in the LAH group was significantly higher than that in the non-LAH group(Wald χ² = 48.317, *P* < 0.001). Specific to different levels of embryos, the blastocyst formation rate (Wald χ² = 6.077, *P* = 0.014) of grade II embryos and the transferable blastocyst formation rate of grade II (Wald χ² = 31.884, *P* < 0.001), II (Wald χ² = 28.226, *P* < 0.001) and IV (Wald χ² = 21.495, *P* < 0.001) embryos in the LAH group were significantly higher than those in the non-LAH group. In addition, the blastocyst formation rate of grade II embryos was also significantly improved in the LAH group (Wald χ² = 6.077, *P* = 0.014). However, there was no significant difference in the high-quality blastocyst formation rate of grade I to IV embryos between the two groups (all *P* > 0.05), and there was no significant difference in other blastocyst rate indexes between the two groups (*P* > 0.05).

**Table 4:** Analysis of the Independent Effects of Laser-Assisted Hatching on Blastocyst Formation Indicators Using Generalized Estimating Equations.

Index	N-LAH	LAH	Wald χ^2^	P	β (95% CI)	SE
Blastocyst formation rate of all cleavage-stage embryos	0.432±0.03	0.457±0.029	3.531	0.06	0.02493 (−0.00107 to 0.05093)	0.01300
Transferable blastocyst formation rate of all cleavage-stage embryos	0.253±0	0.341±0	48.317	<0.001	0.08779 (0.06303 to 0.11254)	0.01300
High-quality blastocyst formation rate of all cleavage-stage embryos	0.144±0.016	0.148±0.015	0.160	0.689	0.00426 (−0.01660 to 0.02511)	0.01100
Blastocyst formation rate of Grade I cleavage-stage embryos	0.688±0.068	0.721±0.067	1.318	0.251	0.03340 (−0.02362 to 0.09043)	0.02900
Transferable blastocyst formation rate of Grade I cleavage-stage embryos	0.565±0.072	0.624±0.069	3.184	0.074	0.05923 (−0.00582 to 0.12428)	0.03300
High-quality blastocyst formation rate of Grade I cleavage-stage embryos	0.403±0.073	0.398±0.07	0.016	0.899	-0.00479 (−0.07841 to 0.06883)	0.03700
Blastocyst formation rate of Grade II cleavage-stage embryos	0.54±0.044	0.586±0.043	6.077	0.014	0.04628 (0.00949 to 0.08308)	0.01900
Transferable blastocyst formation rate of Grade II cleavage-stage embryos	0.363±0.04	0.475±0.039	31.884	<0.001	0.11207 (0.07317 to 0.15096)	0.02000
High-quality blastocyst formation rate of Grade II cleavage-stage embryos	0.227±0.034	0.245±0.033	0.880	0.348	0.01767 (−0.01925 to 0.05459)	0.01900
Blastocyst formation rate of Grade II cleavage-stage embryos	0.486±0.053	0.502±0.052	0.395	0.53	0.01511 (−0.03203 to 0.06224)	0.02400
Transferable blastocyst formation rate of Grade II cleavage-stage embryos	0.291±0.052	0.414±0.051	28.226	<0.001	0.12384 (0.07815 to 0.16952)	0.02300
High-quality blastocyst formation rate of Grade II cleavage-stage embryos	0.201±0.043	0.207±0.041	0.090	0.764	0.00586 (−0.03238 to 0.04409)	0.02000
Blastocyst formation rate of Grade IV cleavage-stage embryos	0.241±0.035	0.253±0.034	0.538	0.463	0.01210 (−0.02023 to 0.04444)	0.01700
Transferable blastocyst formation rate of Grade IV cleavage-stage embryos	0.098±0.027	0.161±0.028	21.495	<0.001	0.06235 (0.03599 to 0.08871)	0.01300
High-quality blastocyst formation rate of Grade IV cleavage-stage embryos	0.045±0.013	0.039±0.012	0.622	0.43	-0.00682 (−0.02376 to 0.01012)	0.00900

### 3.6 Histogram visualization of statistical significance results

**Figure 4:**
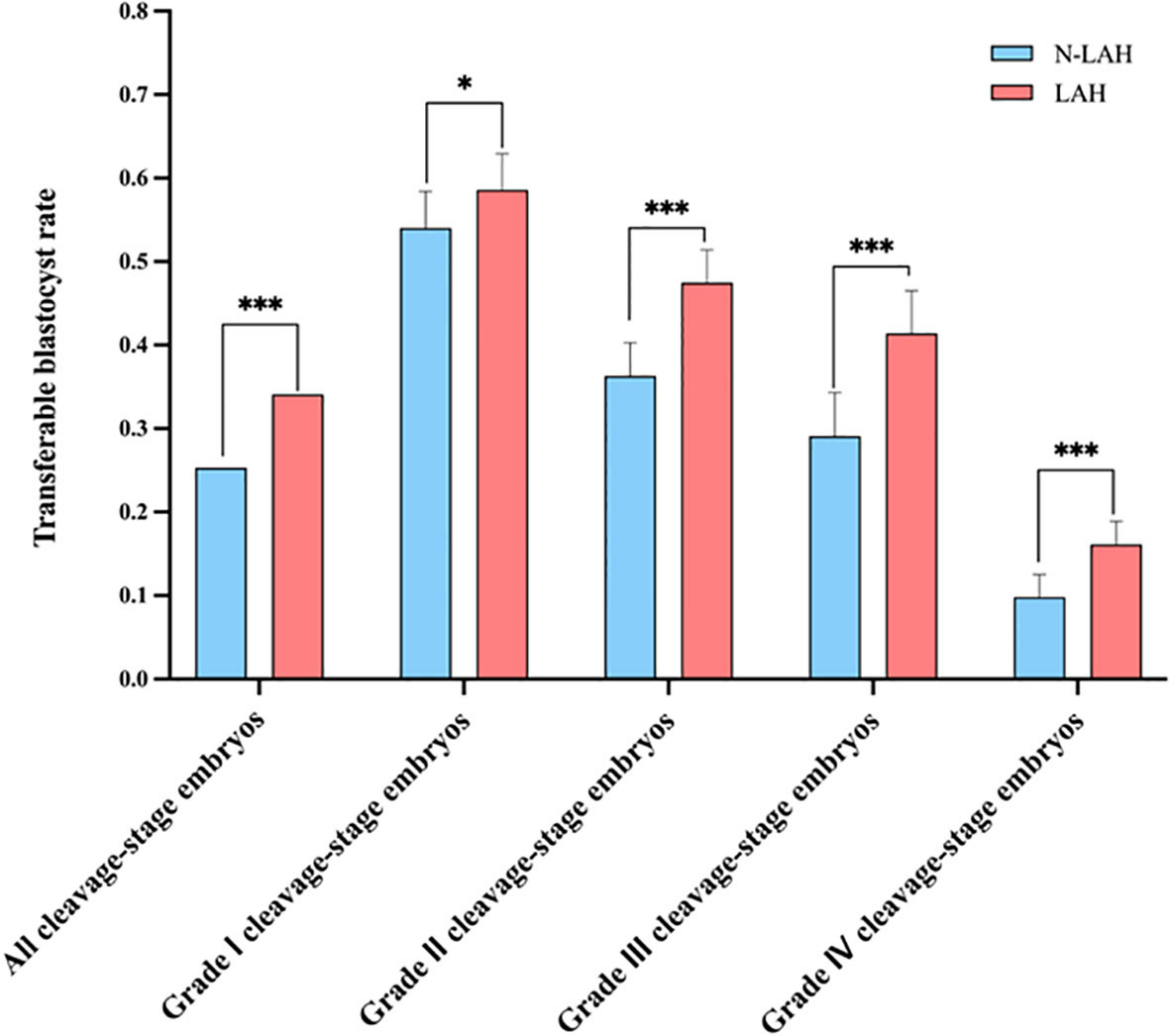
Bar chart of transferable blastocyst rates across cleavage stages (I-IV).

As shown in **Table 4** and **Figure 4**, a histogram is drawn to show the transferable blastocyst rate of different levels of cleavage stage embryos (all cleavage stage embryos, grade I, II, III, and IV cleavage stage embryos) between the LAH and non-LAH groups. As shown in the figure, at all cleavage-stage embryo levels, the rate of ttransferable blastocysts in the LAH group was generally higher than in the non-LAH group, and the difference between groups was statistically significant (marked *, * * *).

As shown in **Table 5**, sensitivity analyses were performed by reconstructing the study cohorts within ±6-month, ± 12-month, and ±24-month calendar-time windows, with additional adjustment for calendar month. Across all models, no significant difference in the overall blastocyst formation rate was observed between the LAH and non-LAH groups (all *P* > 0.05). For transferable blastocyst formation, no significant between-group association was detected in the ±6-month model (*P* > 0.05). However, a positive association was observed in the ±12-month model, with an adjusted difference of 4.62% (*P* = 0.006), and this association became more pronounced in the ±24-month model, with an adjusted difference of 6.69% (*P* < 0.001). For high-quality blastocyst formation, a significant decrease was observed only in the ±6-month model, with an adjusted difference of −6.69% (*P* = 0.008), whereas no consistent pattern was identified in the ±12-month or ±24-month models. Embryo-grade-stratified analyses showed similar trends: the increase in transferable blastocyst formation was mainly observed in the ±12-month and ±24-month models, particularly among grade II–IV embryos, whereas the decrease in high-quality blastocyst formation was detected only in the ±6-month model. Overall, the findings varied according to the selected time window; no evident effect was observed within the ±6-month window, whereas an expanded time window revealed a positive association between LAH and transferable blastocyst formation.

**Table 5:** Time-sensitive analysis (±6, ±12, and ±24 months around LAH initiation date: 2020-05-15; clinical covariates only).

Outcome	N(Total)	LAH(N )	non-LAH(N )	SE	Estimate (95% CI)	P	P formatted	Model df	Condition number	Analysis model
Blastocyst formation rate of all cleavage-stage embryos	600	393	207	0.03116	-0.0268 (-0.0879, 0.0343)	0.390	0.390	14	4.88043	Restricted ±6 months, no time term
	600	393	207	0.05692	-0.0450 (-0.1565, 0.0666)	0.430	0.430	15	8.13015	Restricted ±6 months + month index
Transferable blastocyst formation rate of all cleavage-stage embryos	600	393	207	0.02938	0.0053 (-0.0523, 0.0629)	0.856	0.856	14	4.88043	Restricted ±6 months, no time term
	600	393	207	0.05428	-0.0171 (-0.1235, 0.0893)	0.752	0.752	15	8.13015	Restricted ±6 months + month index
High-quality blastocyst formation rate of all cleavage-stage embryos	600	393	207	0.02513	-0.0669 (-0.1161, -0.0176)	0.008	0.008	14	4.88043	Restricted ±6 months, no time term
	600	393	207	0.04329	-0.1204 (-0.2052, -0.0355)	0.005	0.005	15	8.13015	Restricted ±6 months + month index
Blastocyst formation rate of Grade I cleavage-stage embryos	600	393	207	0.03824	0.0033 (-0.0717, 0.0782)	0.932	0.932	14	4.88043	Restricted ±6 months, no time term
	600	393	207	0.06570	0.0195 (-0.1093, 0.1483)	0.766	0.766	15	8.13015	Restricted ±6 months + month index
Transferable blastocyst formation rate of Grade I cleavage-stage embryos	600	393	207	0.03485	-0.0034 (-0.0718, 0.0649)	0.921	0.921	14	4.88043	Restricted ±6 months, no time term
	600	393	207	0.05854	0.0123 (-0.1024, 0.1271)	0.833	0.833	15	8.13015	Restricted ±6 months + month index
High-quality blastocyst formation rate of Grade I cleavage-stage embryos	600	393	207	0.02742	-0.0269 (-0.0807, 0.0268)	0.326	0.326	14	4.88043	Restricted ±6 months, no time term
	600	393	207	0.04516	-0.0209 (-0.1094, 0.0676)	0.643	0.643	15	8.13015	Restricted ±6 months + month index
Blastocyst formation rate of Grade II cleavage-stage embryos	600	393	207	0.04029	0.0055 (-0.0735, 0.0844)	0.892	0.892	14	4.88043	Restricted ±6 months, no time term
	600	393	207	0.07142	0.0925 (-0.0475, 0.2325)	0.195	0.195	15	8.13015	Restricted ±6 months + month index
Transferable blastocyst formation rate of Grade II cleavage-stage embryos	600	393	207	0.03741	0.0228 (-0.0505, 0.0962)	0.542	0.542	14	4.88043	Restricted ±6 months, no time term
	600	393	207	0.06577	0.0801 (-0.0488, 0.2090)	0.223	0.223	15	8.13015	Restricted ±6 months + month index
High-quality blastocyst formation rate of Grade II cleavage-stage embryos	600	393	207	0.03233	-0.0667 (-0.1301, -0.0034)	0.039	0.039	14	4.88043	Restricted ±6 months, no time term
	600	393	207	0.05256	-0.0820 (-0.1850, 0.0211)	0.119	0.119	15	8.13015	Restricted ±6 months + month index
Blastocyst formation rate of Grade II cleavage-stage embryos	600	393	207	0.03962	0.0373 (-0.0403, 0.1150)	0.346	0.346	14	4.88043	Restricted ±6 months, no time term
	600	393	207	0.06929	0.0120 (-0.1238, 0.1478)	0.862	0.862	15	8.13015	Restricted ±6 months + month index
Transferable blastocyst formation rate of Grade II cleavage-stage embryos	600	393	207	0.03551	0.0472 (-0.0224, 0.1168)	0.184	0.184	14	4.88043	Restricted ±6 months, no time term
	600	393	207	0.06423	-0.0018 (-0.1277, 0.1240)	0.977	0.977	15	8.13015	Restricted ±6 months + month index
High-quality blastocyst formation rate of Grade II cleavage-stage embryos	600	393	207	0.02766	-0.0418 (-0.0960, 0.0125)	0.131	0.131	14	4.88043	Restricted ±6 months, no time term
	600	393	207	0.04819	-0.0794 (-0.1738, 0.0151)	0.100	0.100	15	8.13015	Restricted ±6 months + month index
Blastocyst formation rate of Grade IV cleavage-stage embryos	600	393	207	0.02870	-0.0109 (-0.0671, 0.0454)	0.705	0.705	14	4.88043	Restricted ±6 months, no time term
	600	393	207	0.04864	0.0322 (-0.0631, 0.1276)	0.508	0.508	15	8.13015	Restricted ±6 months + month index
Transferable blastocyst formation rate of Grade IV cleavage-stage embryos	600	393	207	0.02004	0.0139 (-0.0254, 0.0532)	0.488	0.488	14	4.88043	Restricted ±6 months, no time term
	600	393	207	0.03711	0.0508 (-0.0220, 0.1235)	0.171	0.171	15	8.13015	Restricted ±6 months + month index
High-quality blastocyst formation rate of Grade IV cleavage-stage embryos	600	393	207	0.01141	-0.0143 (-0.0366, 0.0081)	0.211	0.211	14	4.88043	Restricted ±6 months, no time term
	600	393	207	0.01468	-0.0141 (-0.0429, 0.0147)	0.337	0.337	15	8.13015	Restricted ±6 months + month index
Blastocyst formation rate of all cleavage-stage embryos	1324	780	544	0.01815	-0.0007 (-0.0363, 0.0349)	0.970	0.970	14	4.29309	Restricted ±12 months, no time term
	1324	780	544	0.03756	-0.0472 (-0.1209, 0.0264)	0.209	0.209	15	7.95648	Restricted ±12 months + month index
Transferable blastocyst formation rate of all cleavage-stage embryos	1324	780	544	0.01696	0.0462 (0.0130, 0.0795)	0.006	0.006	14	4.29309	Restricted ±12 months, no time term
	1324	780	544	0.03579	-0.0108 (-0.0810, 0.0593)	0.762	0.762	15	7.95648	Restricted ±12 months + month index
High-quality blastocyst formation rate of all cleavage-stage embryos	1324	780	544	0.01452	0.0011 (-0.0273, 0.0296)	0.938	0.938	14	4.29309	Restricted ±12 months, no time term
	1324	780	544	0.02969	-0.1099 (-0.1681, -0.0517)	0.000	<0.001	15	7.95648	Restricted ±12 months + month index
Blastocyst formation rate of Grade I cleavage-stage embryos	1324	780	544	0.02281	0.0425 (-0.0022, 0.0872)	0.062	0.062	14	4.29309	Restricted ±12 months, no time term
	1324	780	544	0.04692	-0.0496 (-0.1415, 0.0424)	0.291	0.291	15	7.95648	Restricted ±12 months + month index
Transferable blastocyst formation rate of Grade I cleavage-stage embryos	1324	780	544	0.02125	0.0333 (-0.0084, 0.0749)	0.117	0.117	14	4.29309	Restricted ±12 months, no time term
	1324	780	544	0.04386	-0.0391 (-0.1251, 0.0469)	0.373	0.373	15	7.95648	Restricted ±12 months + month index
High-quality blastocyst formation rate of Grade I cleavage-stage embryos	1324	780	544	0.01742	0.0129 (-0.0212, 0.0470)	0.459	0.459	14	4.29309	Restricted ±12 months, no time term
	1324	780	544	0.03618	-0.0494 (-0.1203, 0.0215)	0.172	0.172	15	7.95648	Restricted ±12 months + month index
Blastocyst formation rate of Grade II cleavage-stage embryos	1324	780	544	0.02430	0.0196 (-0.0280, 0.0673)	0.419	0.419	14	4.29309	Restricted ±12 months, no time term
	1324	780	544	0.04960	-0.0100 (-0.1072, 0.0872)	0.840	0.840	15	7.95648	Restricted ±12 months + month index
Transferable blastocyst formation rate of Grade II cleavage-stage embryos	1324	780	544	0.02284	0.0498 (0.0050, 0.0945)	0.029	0.029	14	4.29309	Restricted ±12 months, no time term
	1324	780	544	0.04570	0.0250 (-0.0646, 0.1146)	0.584	0.584	15	7.95648	Restricted ±12 months + month index
High-quality blastocyst formation rate of Grade II cleavage-stage embryos	1324	780	544	0.01877	0.0032 (-0.0336, 0.0400)	0.865	0.865	14	4.29309	Restricted ±12 months, no time term
	1324	780	544	0.03748	-0.0990 (-0.1725, -0.0256)	0.008	0.008	15	7.95648	Restricted ±12 months + month index
Blastocyst formation rate of Grade II cleavage-stage embryos	1324	780	544	0.02424	0.0242 (-0.0233, 0.0717)	0.318	0.318	14	4.29309	Restricted ±12 months, no time term
	1324	780	544	0.04767	0.0477 (-0.0457, 0.1411)	0.317	0.317	15	7.95648	Restricted ±12 months + month index
Transferable blastocyst formation rate of Grade II cleavage-stage embryos	1324	780	544	0.02078	0.0639 (0.0231, 0.1046)	0.002	0.002	14	4.29309	Restricted ±12 months, no time term
	1324	780	544	0.04305	0.0492 (-0.0352, 0.1335)	0.253	0.253	15	7.95648	Restricted ±12 months + month index
High-quality blastocyst formation rate of Grade II cleavage-stage embryos	1324	780	544	0.01563	0.0122 (-0.0185, 0.0428)	0.436	0.436	14	4.29309	Restricted ±12 months, no time term
	1324	780	544	0.03266	-0.0576 (-0.1216, 0.0064)	0.078	0.078	15	7.95648	Restricted ±12 months + month index
Blastocyst formation rate of Grade IV cleavage-stage embryos	1324	780	544	0.01725	-0.0068 (-0.0406, 0.0270)	0.692	0.692	14	4.29309	Restricted ±12 months, no time term
	1324	780	544	0.03499	0.0170 (-0.0516, 0.0855)	0.628	0.628	15	7.95648	Restricted ±12 months + month index
Transferable blastocyst formation rate of Grade IV cleavage-stage embryos	1324	780	544	0.01301	0.0238 (-0.0017, 0.0493)	0.068	0.068	14	4.29309	Restricted ±12 months, no time term
	1324	780	544	0.02735	0.0431 (-0.0105, 0.0967)	0.115	0.115	15	7.95648	Restricted ±12 months + month index
High-quality blastocyst formation rate of Grade IV cleavage-stage embryos	1324	780	544	0.00772	-0.0038 (-0.0189, 0.0113)	0.623	0.623	14	4.29309	Restricted ±12 months, no time term
	1324	780	544	0.01357	-0.0061 (-0.0327, 0.0205)	0.655	0.655	15	7.95648	Restricted ±12 months + month index
Blastocyst formation rate of all cleavage-stage embryos	2949	1778	1171	0.01206	0.0157 (-0.0079, 0.0394)	0.192	0.192	14	4.38299	Restricted ±24 months, no time term
	2949	1778	1171	0.02516	-0.0294 (-0.0787, 0.0199)	0.242	0.242	15	8.39918	Restricted ±24 months + month index
Transferable blastocyst formation rate of all cleavage-stage embryos	2949	1778	1171	0.01138	0.0669 (0.0446, 0.0892)	0.000	<0.001	14	4.38299	Restricted ±24 months, no time term
	2949	1778	1171	0.02383	0.0198 (-0.0269, 0.0666)	0.405	0.405	15	8.39918	Restricted ±24 months + month index
High-quality blastocyst formation rate of all cleavage-stage embryos	2949	1778	1171	0.00978	0.0025 (-0.0167, 0.0216)	0.801	0.801	14	4.38299	Restricted ±24 months, no time term
	2949	1778	1171	0.01970	-0.0261 (-0.0647, 0.0125)	0.186	0.186	15	8.39918	Restricted ±24 months + month index
Blastocyst formation rate of Grade I cleavage-stage embryos	2949	1778	1171	0.01565	0.0318 (0.0011, 0.0625)	0.042	0.042	14	4.38299	Restricted ±24 months, no time term
	2949	1778	1171	0.03216	0.0499 (-0.0131, 0.1129)	0.121	0.121	15	8.39918	Restricted ±24 months + month index
Transferable blastocyst formation rate of Grade I cleavage-stage embryos	2949	1778	1171	0.01467	0.0323 (0.0035, 0.0610)	0.028	0.028	14	4.38299	Restricted ±24 months, no time term
	2949	1778	1171	0.03007	0.0393 (-0.0196, 0.0983)	0.191	0.191	15	8.39918	Restricted ±24 months + month index
High-quality blastocyst formation rate of Grade I cleavage-stage embryos	2949	1778	1171	0.01264	0.0157 (-0.0091, 0.0404)	0.215	0.215	14	4.38299	Restricted ±24 months, no time term
	2949	1778	1171	0.02428	0.0130 (-0.0346, 0.0605)	0.594	0.594	15	8.39918	Restricted ±24 months + month index
Blastocyst formation rate of Grade II cleavage-stage embryos	2949	1778	1171	0.01685	0.0343 (0.0013, 0.0673)	0.042	0.042	14	4.38299	Restricted ±24 months, no time term
	2949	1778	1171	0.03416	0.0046 (-0.0624, 0.0715)	0.893	0.893	15	8.39918	Restricted ±24 months + month index
Transferable blastocyst formation rate of Grade II cleavage-stage embryos	2949	1778	1171	0.01564	0.0704 (0.0397, 0.1010)	0.000	<0.001	14	4.38299	Restricted ±24 months, no time term
	2949	1778	1171	0.03200	0.0333 (-0.0295, 0.0960)	0.299	0.299	15	8.39918	Restricted ±24 months + month index
High-quality blastocyst formation rate of Grade II cleavage-stage embryos	2949	1778	1171	0.01296	0.0164 (-0.0090, 0.0418)	0.207	0.207	14	4.38299	Restricted ±24 months, no time term
	2949	1778	1171	0.02568	-0.0240 (-0.0744, 0.0263)	0.350	0.350	15	8.39918	Restricted ±24 months + month index
Blastocyst formation rate of Grade II cleavage-stage embryos	2949	1778	1171	0.01650	0.0539 (0.0215, 0.0862)	0.001	0.001	14	4.38299	Restricted ±24 months, no time term
	2949	1778	1171	0.03366	0.0065 (-0.0595, 0.0725)	0.847	0.847	15	8.39918	Restricted ±24 months + month index
Transferable blastocyst formation rate of Grade II cleavage-stage embryos	2949	1778	1171	0.01445	0.0943 (0.0660, 0.1226)	0.000	<0.001	14	4.38299	Restricted ±24 months, no time term
	2949	1778	1171	0.03015	0.0363 (-0.0228, 0.0954)	0.229	0.229	15	8.39918	Restricted ±24 months + month index
High-quality blastocyst formation rate of Grade II cleavage-stage embryos	2949	1778	1171	0.01084	0.0195 (-0.0017, 0.0408)	0.072	0.072	14	4.38299	Restricted ±24 months, no time term
	2949	1778	1171	0.02246	-0.0150 (-0.0591, 0.0290)	0.504	0.504	15	8.39918	Restricted ±24 months + month index
Blastocyst formation rate of Grade IV cleavage-stage embryos	2949	1778	1171	0.01200	0.0024 (-0.0211, 0.0260)	0.840	0.840	14	4.38299	Restricted ±24 months, no time term
	2949	1778	1171	0.02346	-0.0185 (-0.0644, 0.0275)	0.431	0.431	15	8.39918	Restricted ±24 months + month index
Transferable blastocyst formation rate of Grade IV cleavage-stage embryos	2949	1778	1171	0.00920	0.0355 (0.0175, 0.0536)	0.000	<0.001	14	4.38299	Restricted ±24 months, no time term
	2949	1778	1171	0.01844	0.0170 (-0.0191, 0.0532)	0.356	0.356	15	8.39918	Restricted ±24 months + month index
High-quality blastocyst formation rate of Grade IV cleavage-stage embryos	2949	1778	1171	0.00572	-0.0055 (-0.0167, 0.0057)	0.335	0.335	14	4.38299	Restricted ±24 months, no time term
	2949	1778	1171	0.01000	-0.0064 (-0.0260, 0.0132)	0.523	0.523	15	8.39918	Restricted ±24 months + month index

## 4 Discussion

Based on a single center large sample retrospective cohort (including 4365 couples), this study focused on the application value and controversy of LAH in ART, using PSM to assess the key confounding factors such as age, BMI, infertility duration, baseline hormone levels, and ovulation induction protocol were balanced for the first time (After matching, there was no statistical difference between all variable groups. *P* > 0.05). GEE models were applied to evaluate the association between LAH and blastocyst formation after adjustment for measured confounders, including fresh D3 cleavage-stage embryos and blastocysts of different quality grades (I–IV). This approach reduces common selection bias and confounding inherent to retrospective studies, providing more reliable evidence for the clinical interpretation of LAH effects.

The research results show that age is a key factor in embryonic development potential. Younger patients (regardless of gender) have significantly higher blastocyst formation rates, transferable blastocyst rates, and high-quality blastocyst rates than the older group (all *P* < 0.05), especially in grade I and II embryos. These findings indicate an association between advanced parental age and reduced embryonic developmental indicators, consistent with previously reported trends. The underlying biological mechanisms, including potential impacts on oocyte quality or mitochondrial function, were not directly assessed in this study and therefore remain speculative. Female obesity (BMI ≥30 kg/m²) was associated with lower blastocyst formation, transferable blastocyst formation, and high-quality blastocyst formation rates among Grade II embryos (P = 0.031, 0.005, and 0.028, respectively), whereas paternal BMI showed no significant effect. These findings indicate that maternal metabolic and endocrine status may play a larger role in early embryonic development (19). In addition, a systematic review has indicated that women aged 38 years or older, obese, and with repeated implantation failures are associated with a decrease in blastocyst ploidy rate (20), which could provide potential genetic support for the observed reduction in blastocyst developmental indicators among older and obese patients in this study.

In addition to age and BMI, this study systematically revealed the differential effects of infertility type, ovulation induction protocol, infertility duration, and endocrine indicators on embryonic development potential. Multiple regression analysis showed that patients with primary infertility had higher blastocyst formation, transferable blastocyst formation, and high-quality blastocyst formation rates among grade III embryos than patients with secondary infertility. These findings suggest that infertility type may be associated with embryo developmental outcomes; however, the underlying mechanisms could not be evaluated in the present study. Conventional stimulation protocols, including long and antagonist regimens, were associated with more favorable blastocyst developmental indicators than mild stimulation protocols. This observation may partially reflect differences in patient characteristics across stimulation strategies, as mild stimulation protocols are frequently used in women with diminished ovarian reserve (21). Therefore, the observed differences should not be interpreted as evidence of a direct effect of stimulation protocol itself on embryo developmental competence. Infertility duration showed weak negative correlations with several blastocyst developmental outcomes (**Table 3**). These findings support the established association between prolonged infertility duration and impaired embryo developmental competence (22, 23), the observed correlation coefficients were relatively small, and most associations were attenuated after correction for multiple testing. Therefore, the clinical significance of these correlations should be interpreted cautiously. In terms of basic endocrine function, basal FSH is positively correlated with the blastocyst formation rate (Rs = 0.121, *P* = 0.007) and transferable rate (Rs = 0.116, *P* = 0.009) of grade I embryos, while negatively correlated with the high-quality rate of grade IV embryos (Rs = -0.059, *P* = 0.019), indicating a limited association rather than a major determinant of blastocyst development. The basic AMH is positively correlated with the transferable rate (Rs = 0.045, *P* = 0.047) and high-quality rate (Rs = 0.114, *P* < 0.001) of all cleavage embryos. However, the magnitude of these correlations remained modest, suggesting that AMH may contribute to variability in embryonic developmental outcomes but should not be regarded as an independent predictor of blastocyst developmental potential based on the present data alone (24). In addition, basal LH has a positive regulatory effect on the total high-quality blastocyst rate (Rs = 0.090, *P* < 0.001). In contrast, basal PRL inhibits the blastocyst formation rate of grade III embryos (Rs = -0.061, *P* = 0.035). Because these associations are small in magnitude, their biological significance remains uncertain and warrants further investigation. No significant association was observed between basal E_2_ levels and blastocyst developmental outcomes in the present cohort.

After controlling for the above confounding factors, GEE multivariate adjustment showed that the transferable blastocyst rate in all embryos and grade II, III, and IV embryos in the LAH group was significantly higher than that in the non-LAH group (all *P* < 0.001). At the same time, there was no intergroup difference in high-quality blastocyst rate (*P* > 0.05, **Table 4**). The lack of difference in the rate of high-quality blastocysts may be related to conservative scoring criteria (such as strictly limiting to Gardner AA grade), resulting in some blastocysts with developmental potential being classified as non-high-quality, rather than to ineffective LAH technology itself. This result is broadly consistent with the report by Xu et al. (25), supporting the potential benefit of LAH in improving blastocyst formation among low-grade cleavage embryos. However, differences in study design, patient characteristics, embryo selection criteria, and outcome definitions should be considered when comparing findings across studies. From a mechanistic perspective, LAH reduces mechanical resistance and assists low-quality embryos in overcoming hatching barriers by laser-thinning the zona pellucida (parameters in this study: 1480 nm, aperture 4.0-6.9 μm); however, high-quality embryos have strong self-hatching ability, and the additional benefits of LAH are limited. Previous prospective studies have reported heterogeneous results regarding assisted hatching, including the work of Sagoskin et al. (26), without providing definitive conclusions on blastocyst developmental outcomes. In addition, a large-scale retrospective study by Wei et al. (27) showed that LAH can improve the live birth rate of frozen-thawed cleavage embryos. At the same time, the ALADDIN test of Alteri et al. (28) found that LAH did not significantly improve the live birth rate of frozen-thawed embryos. The possible reason for the difference between the two is the embryonic developmental stage: cleavage embryos have a thicker zona pellucida and weaker hatching ability, and the LAH intervention value is therefore more significant. However, the blastocysts have partially hatched, and the zona pellucida resistance is reduced. The additional benefits of LAH may be limited, further supporting the rationality and clinical significance of this study focusing on fresh D3 cleavage embryos.

In this large retrospective cohort, the multi-window time sensitivity analyses showed heterogeneous findings across the ±6-month, ± 12-month, and ±24-month models. The overall blastocyst formation rate remained unchanged across all time-window models (*P* > 0.05). However, the transferable blastocyst formation rate was significantly increased in the ±12-month model, with an adjusted difference of 4.62% (*P* = 0.006), and this increase became more pronounced in the ±24-month model, with an adjusted difference of 6.69% (*P* < 0.001). This potential benefit was mainly observed among grade II–IV embryos. In contrast, the high-quality blastocyst formation rate decreased only in the ±6-month model, with an adjusted difference of −6.69% (*P* = 0.008), whereas no consistent trend was observed over longer observation windows. These findings suggest that LAH may have a relatively stable association with improved embryo transferability, particularly among lower-grade cleavage-stage embryos, but its effect on high-quality blastocyst formation appears limited.

Embryos were cultured under standardized laboratory conditions throughout the study period. Commercial PLUS series media from Vitrolife, Sweden, including G-IVF™ PLUS, G-1™ PLUS, G-2™ PLUS, and G-MOPS™ PLUS, were used for embryo handling and culture. The incubator parameters were maintained at a constant level, with the temperature controlled at 37.0 ± 0.1 °C, CO_2_ concentration at 6.0% ± 0.2%, and O_2_ concentration at 5.0% ± 0.2%. These environmental parameters were continuously monitored in accordance with the laboratory quality-control procedures. LAH was performed using a standardized non-contact infrared diode laser system (ZILOS-tk; Hamilton Thorne Biosciences, USA) combined with an Evident IX73P1F inverted microscope (formerly Olympus, Japan). All LAH procedures were conducted by senior embryologists with more than 3 years of micromanipulation experience and were performed strictly according to a unified standard operating procedure. The standardized management of operators, equipment, culture conditions, and laboratory workflow helped minimize technical variation and reduce potential experimental bias.

Although there is heterogeneity in the clinical benefits of LAH in the existing literature, these heterogeneities are closely related to factors such as research subjects, embryonic stage, transplantation type, and technical pathway. Xu et al. (2023) (29) adopted a sequential strategy of “D4 first LAH combined with D6 second LAH”, which significantly improved the D7 blastocyst formation rate of low-quality embryos and, together with the findings of the present study, suggests that LAH may be associated with improved developmental outcomes in selected low-grade embryos. The meta-analysis by Chen et al. (2024) (2) did not clearly determine the absolute superiority or inferiority of D-LAH and T-LAH. Similarly, the present study observed an association between D-LAH and blastocyst developmental outcomes in fresh D3 cleavage-stage embryos under specific analytical conditions. Lu et al. (2019) (30) reported that LAH can improve the outcome of freeze-thaw cycles in patients with repeated transplant failures, while the multicenter RCT of Curfs et al. (2023) (10) did not show any improvement in cumulative live birth rate with LAH, suggesting that the observed effects of LAH may be regulated by both the patient’s underlying etiology and embryonic status. Jiang et al. (2025) (13) found that LAH specifically enhances the implantation potential of grade TE-C blastocysts, which is generally consistent with the observation that the associations identified in the present study were more evident among embryos with relatively lower developmental potential.

Based on the above studies, it can be seen that the reported effects of LAH are not consistent across studies and may vary according to embryo quality (low rating vs high quality), developmental stage (cleavage vs blastocyst), transplantation type (fresh vs freeze-thaw), patient characteristics (age, infertility causes, endometrial preparation plan), and technical pathway (D-LAH vs T-LAH, single vs secondary). The core of the current controversy lies in the lack of standardized research designs targeting specific populations. This study focused on the blastocyst formation stage of fresh D3 cleavage embryos and did not extend to the evaluation of pregnancy outcomes and offspring safety. Therefore, further exploration is needed to investigate how LAH improves implantation and live birth rates after blastocyst formation; the long-term benefit difference between D-LAH and T-LAH in low-quality cleavage embryos; and personalized application thresholds for specific populations, such as advanced age and repeated failures.

These findings may inform considerations for the application of LAH in different embryo grades. For grade I high-quality fresh D3 cleavage embryos, conventional LAH is not necessary and can avoid unnecessary embryo intervention; For grade II-IV low-quality fresh D3 cleavage embryos, LAH can significantly improve the transferable blastocyst rate and increase the number of available embryos for patients, especially for patients with poor ovarian reserve and limited available embryos, providing an effective embryo rescue solution for the ART treatment cycle. But the clinical significance of these associations requires further investigation.

Several limitations should be acknowledged. First, this was a single-center retrospective study. Although PSM and GEE models were used to adjust for measured confounders, residual and unmeasured confounding could not be completely excluded. Second, ICSI cycles were excluded from the present analysis; therefore, extrapolation of these findings to the broader ART population should be performed with caution. Third, the present study evaluated only embryological laboratory outcomes, including blastocyst formation rate, transferable blastocyst formation rate, and high-quality blastocyst formation rate. Subsequent clinical outcomes, including embryo transfer outcomes, implantation rate, clinical pregnancy rate, miscarriage rate, live birth rate, twin pregnancy rate, monozygotic twinning rate, neonatal outcomes, and long-term offspring safety, were not systematically collected or analyzed. Therefore, the present findings should be interpreted as embryological observational data and should not be considered direct evidence of the clinical effectiveness or safety of LAH. Future prospective studies with standardized reproductive and neonatal follow-up are needed to determine whether LAH improves clinically meaningful outcomes, particularly live birth and offspring safety.

There are several limitations to this study: as a single-center retrospective study, although PSM-GEE controlled for known confounders, there may still be unmeasured bias; excluding patients with ICSI cycles means extrapolation should be done cautiously, which aligns with the high heterogeneity and low quality of evidence in LAH studies, as noted in the Ge et al. review (14). The study did not evaluate pregnancy outcomes and offspring safety; the D-LAH approach may increase the risk of multiple pregnancies, but the relevant research evidence is not yet sufficient; in clinical practice, it is important to fully inform patients about this potential risk. In particular, “8-shaped” hatching from a small zona opening may cause mechanical restriction or compression of the inner cell mass during blastocyst herniation, thereby theoretically increasing the risk of inner cell mass splitting. From an embryological perspective, atypical hatching morphologies, such as “8-shaped” or incompletely separated hatching, have been considered a potential mechanism underlying MZT. When a blastocyst hatches through a small artificial zona opening, the inner cell mass may theoretically be compressed or mechanically divided during herniation, thereby increasing the possibility of inner cell mass splitting. In the present study, LAH was performed using a standardized non-contact infrared diode laser system, and only a small zona opening was created; however, the potential influence of zona manipulation on blastocyst hatching morphology could not be completely excluded. Because pregnancy follow-up data were not systematically collected, twin pregnancy rate and MZT incidence could not be analyzed in this study. Therefore, this safety issue should be fully explained to patients in clinical practice. Future prospective studies incorporating time-lapse embryo imaging and long-term reproductive follow-up are needed to determine whether LAH-related atypical hatching morphology is associated with an increased risk of twin pregnancy or MZT.

To summarize, this study confirmed, using PSM-GEE multivariate correction, that LAH was independent of age, BMI, infertility duration, baseline hormone levels, and other factors. It significantly increased the transferable blastocyst rate of low-grade (II-IV) fresh D3 cleavage embryos, providing an evidence-based foundation for applying LAH in the fresh cycle of low-quality cleavage embryos. Moving forward, a multicenter, prospective RCT is needed to further verify its effects on implantation rate, live birth rate, and offspring safety. However, the time-sensitivity analyses yielded heterogeneous findings across different observation windows, and the observed associations were attenuated after adjustment for calendar time, suggesting that temporal factors may have contributed to the results. Therefore, these findings should be interpreted cautiously.

## 5 Conclusion

In this large single-center retrospective cohort, the independent effect of LAH on blastocyst development from fresh D3 cleavage-stage embryos was evaluated using PSM combined with GEE modeling. After rigorous adjustment for key confounders, including age, BMI, infertility duration, baseline endocrine profiles, infertility type, and stimulation protocols, LAH was shown to significantly increase the rate of transferable blastocysts, particularly among low-grade (II–IV) cleavage-stage embryos, while no improvement was observed in high-quality blastocyst rates. These findings indicated that LAH primarily exerted a rescue effect on embryos with limited developmental potential rather than enhancing the competence of high-quality embryos. Furthermore, embryo developmental outcomes were strongly associated with patient-related factors, with AMH showing consistent positive correlations, especially in low-grade embryos. These results support a selective rather than routine application of LAH in ART, favoring its use in fresh D3 low-grade cleavage embryos, particularly in patients with limited embryo availability. However, this study was limited to embryological laboratory outcomes and did not systematically evaluate subsequent embryo transfer outcomes, implantation rate, clinical pregnancy rate, miscarriage rate, live birth rate, twin pregnancy rate, monozygotic twinning rate, neonatal outcomes, or long-term offspring safety. Therefore, the present findings should be interpreted cautiously and should not be considered direct evidence of the clinical effectiveness or safety of LAH. Prospective multicenter randomized controlled trials with standardized reproductive and neonatal follow-up are warranted to further validate the effects of LAH on implantation, live birth, and offspring safety.

## Data Availability

The datasets presented in this study can be found in online repositories. The names of the repository/repositories and accession number(s) can be found in the article/supplementary material.
