# Supplementary material for: Association between laser-assisted hatching and subsequent blastocyst development in fresh day 3 cleavage-stage embryos: a retrospective cohort study using propensity score matching, generalized estimating equations, and time-sensitivity analyses
Source: Front Endocrinol (Lausanne). 2026 Jul 8;17:1871377. doi: 10.3389/fendo.2026.1871377 (PMC13388058; doi:10.3389/fendo.2026.1871377)
Supplement: Supplementary file 1 [file Table1.docx]

**Table S1.** Subgroup analysis of blastocyst development outcomes by female partner's age

| Outcome measure | <35 (n=1628) Median (IQR) | 35–39 (n=290) Median (IQR) | >39 (n=80) Median (IQR) | H | η² (95% CI) | P value | Adjusted P value† | ρ | P for trend |
| --- | --- | --- | --- | --- | --- | --- | --- | --- | --- |
| Blastocyst formation, all stages | 0.5 (0.308, 0.714) | 0.5 (0.302, 0.667) | 0.333 (0, 0.525) | 17.155 | 0.008 (0.002, 0.018) | <0.001 | <0.001 | -0.063 | 0.005 |
| Transferable blastocyst, all stages | 0.333 (0.151, 0.571) | 0.333 (0.157, 0.5) | 0 (0, 0.339) | 30.745 | 0.014 (0.006, 0.031) | <0.001 | <0.001 | -0.076 | <0.001 |
| High-quality blastocyst, all stages | 0.167 (0, 0.333) | 0.1 (0, 0.296) | 0 (0, 0) | 42.714 | 0.02 (0.011, 0.03) | <0.001 | <0.001 | -0.116 | <0.001 |
| Blastocyst formation, Grade I | 1 (0.667, 1) | 1 (0.667, 1) | 0.633 (0.281, 1) | 6.096 | 0.008 (0, 0.041) | 0.047 | 0.055 | -0.08 | 0.076 |
| Transferable blastocyst, Grade I | 1 (0.5, 1) | 1 (0.5, 1) | 0.388 (0, 1) | 5.589 | 0.007 (0, 0.038) | 0.061 | 0.066 | -0.072 | 0.108 |
| High-quality blastocyst, Grade I | 0.5 (0, 1) | 0.333 (0, 1) | 0 (0, 0.467) | 9.96 | 0.016 (0.001, 0.044) | 0.007 | 0.013 | -0.135 | 0.003 |
| Blastocyst formation, Grade II | 0.75 (0.5, 1) | 0.714 (0.5, 1) | 0.5 (0, 1) | 8.661 | 0.005 (0, 0.017) | 0.013 | 0.022 | -0.054 | 0.048 |
| Transferable blastocyst, Grade II | 0.571 (0.333, 1) | 0.5 (0.225, 1) | 0.5 (0, 0.667) | 6.163 | 0.003 (0, 0.016) | 0.046 | 0.055 | -0.038 | 0.166 |
| High-quality blastocyst, Grade II | 0.3 (0, 0.5) | 0 (0, 0.5) | 0 (0, 0.317) | 15.911 | 0.01 (0.003, 0.026) | <0.001 | 0.001 | -0.102 | <0.001 |
| Blastocyst formation, Grade III | 0.667 (0, 1) | 0.5 (0, 1) | 0.333 (0, 1) | 4.878 | 0.002 (0, 0.01) | 0.087 | 0.087 | -0.057 | 0.047 |
| Transferable blastocyst, Grade III | 0.4 (0, 1) | 0.2 (0, 0.667) | 0 (0, 0.5) | 12.158 | 0.008 (0.002, 0.022) | 0.002 | 0.006 | -0.094 | 0.001 |
| High-quality blastocyst, Grade III | 0 (0, 0.333) | 0 (0, 0.333) | 0 (0, 0) | 7.233 | 0.004 (0, 0.012) | 0.027 | 0.040 | -0.067 | 0.020 |
| Blastocyst formation, Grade IV | 0.2 (0, 0.5) | 0.15 (0, 0.5) | 0 (0, 0.25) | 13.412 | 0.007 (0.003, 0.017) | 0.001 | 0.004 | -0.065 | 0.009 |
| Transferable blastocyst, Grade IV | 0 (0, 0.25) | 0 (0, 0.25) | 0 (0, 0) | 11.07 | 0.006 (0.001, 0.015) | 0.004 | 0.008 | -0.062 | 0.014 |
| High-quality blastocyst, Grade IV | 0 (0, 0) | 0 (0, 0) | 0 (0, 0) | 6.165 | 0.003 (0, 0.007) | 0.046 | 0.055 | -0.036 | 0.150 |

Note: Data are presented as median (interquartile range, IQR). Group comparisons performed using Kruskal–Wallis H test.

† P values adjusted using the Benjamini-Hochberg false discovery rate (FDR) procedure within each variable.

P for trend calculated using Spearman rank correlation test.

IQR: interquartile range; CI: confidence interval.
