# Supplementary material for: Association between laser-assisted hatching and subsequent blastocyst development in fresh day 3 cleavage-stage embryos: a retrospective cohort study using propensity score matching, generalized estimating equations, and time-sensitivity analyses
Source: Front Endocrinol (Lausanne). 2026 Jul 8;17:1871377. doi: 10.3389/fendo.2026.1871377 (PMC13388058; doi:10.3389/fendo.2026.1871377)
Supplement: Supplementary file 2 [file Table2.docx]

**Table S2.** Subgroup analysis of blastocyst development outcomes by male partner's age.

| Outcome measure | <35 (n=1451) Median (IQR) | 35–39 (n=378) Median (IQR) | >39 (n=169) Median (IQR) | H | η² (95% CI) | P value | Adjusted P value† | ρ | P for trend |
| --- | --- | --- | --- | --- | --- | --- | --- | --- | --- |
| Blastocyst formation, all stages | 0.5 (0.308, 0.714) | 0.5 (0.302, 0.667) | 0.5 (0.182, 0.667) | 5.35 | 0.002 (0, 0.009) | 0.069 | 0.129 | -0.047 | 0.035 |
| Transferable blastocyst, all stages | 0.333 (0.154, 0.568) | 0.333 (0.128, 0.571) | 0.3 (0, 0.5) | 6.68 | 0.002 (0, 0.011) | 0.035 | 0.086 | -0.038 | 0.088 |
| High-quality blastocyst, all stages | 0.167 (0, 0.333) | 0.125 (0, 0.333) | 0 (0, 0.25) | 16.154 | 0.007 (0.002, 0.017) | <0.001 | 0.005 | -0.072 | 0.001 |
| Blastocyst formation, Grade I | 1 (0.667, 1) | 1 (0.762, 1) | 1 (0.365, 1) | 6.432 | 0.009 (0, 0.04) | 0.040 | 0.086 | -0.043 | 0.342 |
| Transferable blastocyst, Grade I | 1 (0.5, 1) | 1 (0.617, 1) | 0.5 (0, 1) | 8.606 | 0.013 (0.001, 0.044) | 0.014 | 0.041 | -0.026 | 0.567 |
| High-quality blastocyst, Grade I | 0.5 (0, 1) | 0.55 (0, 1) | 0 (0, 0.5) | 11.592 | 0.019 (0.001, 0.041) | 0.003 | 0.023 | -0.083 | 0.065 |
| Blastocyst formation, Grade II | 0.75 (0.5, 1) | 0.75 (0.5, 1) | 0.667 (0, 1) | 4.023 | 0.002 (0, 0.012) | 0.134 | 0.201 | -0.029 | 0.287 |
| Transferable blastocyst, Grade II | 0.571 (0.333, 1) | 0.6 (0.31, 1) | 0.5 (0, 1) | 3.568 | 0.001 (0, 0.017) | 0.168 | 0.229 | -0.022 | 0.423 |
| High-quality blastocyst, Grade II | 0.286 (0, 0.5) | 0.25 (0, 0.5) | 0 (0, 0.5) | 10.51 | 0.006 (0.000123, 0.019) | 0.005 | 0.026 | -0.071 | 0.009 |
| Blastocyst formation, Grade III | 0.667 (0, 1) | 0.667 (0, 1) | 0.5 (0, 1) | 2.199 | 0.000166 (0, 0.009) | 0.333 | 0.363 | -0.034 | 0.237 |
| Transferable blastocyst, Grade III | 0.333 (0, 1) | 0.4 (0, 1) | 0.1 (0, 0.75) | 2.654 | 0.001 (0, 0.008) | 0.265 | 0.332 | -0.019 | 0.518 |
| High-quality blastocyst, Grade III | 0 (0, 0.333) | 0 (0, 0.333) | 0 (0, 0.25) | 0.932 | 0 (0, 0.008) | 0.628 | 0.628 | -0.027 | 0.342 |
| Blastocyst formation, Grade IV | 0.222 (0, 0.5) | 0.05 (0, 0.5) | 0 (0, 0.5) | 9.621 | 0.005 (0.00015, 0.019) | 0.008 | 0.031 | -0.078 | 0.002 |
| Transferable blastocyst, Grade IV | 0 (0, 0.286) | 0 (0, 0.25) | 0 (0, 0.211) | 4.953 | 0.002 (0, 0.008) | 0.084 | 0.140 | -0.056 | 0.027 |
| High-quality blastocyst, Grade IV | 0 (0, 0) | 0 (0, 0) | 0 (0, 0) | 2.165 | 0.000104 (0, 0.005) | 0.339 | 0.363 | -0.009 | 0.728 |

Note: Data are presented as median (interquartile range, IQR). Group comparisons performed using Kruskal–Wallis H test.

† P values adjusted using the Benjamini-Hochberg false discovery rate (FDR) procedure within each variable.

P for trend calculated using Spearman rank correlation test.

IQR: interquartile range; CI: confidence interval.
