# Supplementary material for: Association between laser-assisted hatching and subsequent blastocyst development in fresh day 3 cleavage-stage embryos: a retrospective cohort study using propensity score matching, generalized estimating equations, and time-sensitivity analyses
Source: Front Endocrinol (Lausanne). 2026 Jul 8;17:1871377. doi: 10.3389/fendo.2026.1871377 (PMC13388058; doi:10.3389/fendo.2026.1871377)
Supplement: Supplementary file 3 [file Table3.docx]

**Table S3.** Subgroup analysis of blastocyst development outcomes by female partner's BMI.

| Outcome measure | 18.5–24.9 (n=1638) Median (IQR) | 25.0–29.9 (n=334) Median (IQR) | ≥30 (n=26) Median (IQR) | H | η² (95% CI) | P value | Adjusted P value† | ρ | P for trend |
| --- | --- | --- | --- | --- | --- | --- | --- | --- | --- |
| Blastocyst formation, all stages | 0.5 (0.308, 0.7) | 0.5 (0.25, 0.689) | 0.389 (0.05, 0.619) | 3.67 | 0.001 (0, 0.01) | 0.160 | 0.342 | -0.041 | 0.066 |
| Transferable blastocyst, all stages | 0.333 (0.143, 0.571) | 0.333 (0.143, 0.522) | 0.2 (0, 0.4) | 5.62 | 0.002 (0, 0.01) | 0.060 | 0.226 | -0.036 | 0.110 |
| High-quality blastocyst, all stages | 0.143 (0, 0.333) | 0.143 (0, 0.333) | 0 (0, 0.186) | 4.709 | 0.001 (0, 0.006) | 0.095 | 0.285 | -0.024 | 0.287 |
| Blastocyst formation, Grade I | 1 (0.667, 1) | 1 (0.688, 1) | 1 (0.625, 1) | 0.278 | 0 (0, 0.018) | 0.870 | 0.870 | 0.014 | 0.762 |
| Transferable blastocyst, Grade I | 1 (0.5, 1) | 1 (0.5, 1) | 0.75 (0.375, 1) | 0.688 | 0 (0, 0.018) | 0.709 | 0.814 | -0.034 | 0.445 |
| High-quality blastocyst, Grade I | 0.5 (0, 1) | 0.5 (0, 0.938) | 0.25 (0, 0.875) | 0.829 | 0 (0, 0.015) | 0.661 | 0.814 | -0.039 | 0.389 |
| Blastocyst formation, Grade II | 0.75 (0.5, 1) | 0.786 (0.5, 1) | 0.583 (0, 0.779) | 4.189 | 0.002 (0, 0.011) | 0.123 | 0.308 | -0.008 | 0.773 |
| Transferable blastocyst, Grade II | 0.571 (0.25, 1) | 0.5 (0.333, 1) | 0 (0, 0.405) | 10.602 | 0.006 (0.001, 0.017) | 0.005 | 0.075 | -0.007 | 0.785 |
| High-quality blastocyst, Grade II | 0.25 (0, 0.5) | 0.333 (0, 0.5) | 0 (0, 0.125) | 7.134 | 0.004 (0, 0.011) | 0.028 | 0.155 | -0.004 | 0.875 |
| Blastocyst formation, Grade III | 0.667 (0, 1) | 0.5 (0, 1) | 0.5 (0, 1) | 0.863 | 0 (0, 0.008) | 0.650 | 0.814 | -0.027 | 0.353 |
| Transferable blastocyst, Grade III | 0.333 (0, 1) | 0.333 (0, 0.667) | 0.5 (0, 1) | 2.546 | 0.000455 (0, 0.008) | 0.280 | 0.490 | -0.04 | 0.162 |
| High-quality blastocyst, Grade III | 0 (0, 0.333) | 0 (0, 0.321) | 0 (0, 0.5) | 1.066 | 0 (0, 0.005) | 0.587 | 0.814 | -0.023 | 0.418 |
| Blastocyst formation, Grade IV | 0.214 (0, 0.5) | 0 (0, 0.4) | 0 (0, 0.5) | 6.954 | 0.003 (0, 0.012) | 0.031 | 0.155 | -0.065 | 0.009 |
| Transferable blastocyst, Grade IV | 0 (0, 0.25) | 0 (0, 0.25) | 0 (0, 0) | 2.447 | 0.000283 (0, 0.007) | 0.294 | 0.490 | -0.027 | 0.277 |
| High-quality blastocyst, Grade IV | 0 (0, 0) | 0 (0, 0) | 0 (0, 0) | 0.549 | 0 (0, 0.003) | 0.760 | 0.814 | 0.001 | 0.978 |

Note: Data are presented as median (interquartile range, IQR). Group comparisons performed using Kruskal–Wallis H test.

† P values adjusted using the Benjamini-Hochberg false discovery rate (FDR) procedure within each variable.

P for trend calculated using Spearman rank correlation test.

IQR: interquartile range; CI: confidence interval.
