# Supplementary material for: Association between laser-assisted hatching and subsequent blastocyst development in fresh day 3 cleavage-stage embryos: a retrospective cohort study using propensity score matching, generalized estimating equations, and time-sensitivity analyses
Source: Front Endocrinol (Lausanne). 2026 Jul 8;17:1871377. doi: 10.3389/fendo.2026.1871377 (PMC13388058; doi:10.3389/fendo.2026.1871377)
Supplement: Supplementary file 4 [file Table4.docx]

**Table S4.** Subgroup analysis of blastocyst development outcomes by male partner's BMI.

| Outcome measure | 18.5–24.9 (n=1178) Median (IQR) | 25.0–29.9 (n=703) Median (IQR) | ≥30 (n=117) Median (IQR) | H | η² (95% CI) | P value | Adjusted P value† | ρ | P for trend |
| --- | --- | --- | --- | --- | --- | --- | --- | --- | --- |
| Blastocyst formation, all stages | 0.5 (0.333, 0.714) | 0.5 (0.25, 0.686) | 0.5 (0.333, 0.667) | 3.61 | 0.001 (0, 0.008) | 0.164 | 0.617 | -0.03 | 0.180 |
| Transferable blastocyst, all stages | 0.333 (0.167, 0.571) | 0.333 (0.087, 0.556) | 0.333 (0, 0.571) | 3.808 | 0.001 (0, 0.005) | 0.149 | 0.617 | -0.04 | 0.072 |
| High-quality blastocyst, all stages | 0.167 (0, 0.333) | 0.111 (0, 0.333) | 0.143 (0, 0.333) | 3.81 | 0.001 (0, 0.008) | 0.149 | 0.617 | -0.034 | 0.129 |
| Blastocyst formation, Grade I | 1 (0.708, 1) | 1 (0.5, 1) | 1 (1, 1) | 1.51 | 0 (0, 0.018) | 0.470 | 0.826 | -0.031 | 0.483 |
| Transferable blastocyst, Grade I | 1 (0.5, 1) | 1 (0.5, 1) | 1 (0.542, 1) | 1.121 | 0 (0, 0.016) | 0.571 | 0.826 | -0.001 | 0.982 |
| High-quality blastocyst, Grade I | 0.5 (0, 1) | 0.5 (0, 1) | 0.69 (0.375, 1) | 1.972 | 0 (0, 0.023) | 0.373 | 0.799 | 0.006 | 0.897 |
| Blastocyst formation, Grade II | 0.75 (0.5, 1) | 0.75 (0.5, 1) | 0.8 (0.5, 1) | 1.225 | 0 (0, 0.005) | 0.542 | 0.826 | -0.025 | 0.368 |
| Transferable blastocyst, Grade II | 0.556 (0.25, 1) | 0.5 (0.25, 1) | 0.667 (0.25, 1) | 2.275 | 0.000204 (0, 0.006) | 0.321 | 0.799 | -0.032 | 0.242 |
| High-quality blastocyst, Grade II | 0.25 (0, 0.5) | 0.25 (0, 0.5) | 0.25 (0, 0.667) | 0.956 | 0 (0, 0.007) | 0.620 | 0.826 | -0.007 | 0.802 |
| Blastocyst formation, Grade III | 0.667 (0, 1) | 0.5 (0, 1) | 0.667 (0, 1) | 0.236 | 0 (0, 0.005) | 0.889 | 0.889 | -0.012 | 0.673 |
| Transferable blastocyst, Grade III | 0.333 (0, 1) | 0.333 (0, 1) | 0.5 (0, 1) | 0.588 | 0 (0, 0.006) | 0.745 | 0.826 | 0.001 | 0.965 |
| High-quality blastocyst, Grade III | 0 (0, 0.333) | 0 (0, 0.333) | 0 (0, 0.5) | 4.859 | 0.002 (0, 0.015) | 0.088 | 0.617 | 0.051 | 0.077 |
| Blastocyst formation, Grade IV | 0.2 (0, 0.5) | 0.167 (0, 0.5) | 0.25 (0, 0.5) | 2.221 | 0.00014 (0, 0.006) | 0.329 | 0.799 | -0.011 | 0.665 |
| Transferable blastocyst, Grade IV | 0 (0, 0.25) | 0 (0, 0.25) | 0 (0, 0.333) | 0.756 | 0 (0, 0.005) | 0.685 | 0.826 | -0.008 | 0.750 |
| High-quality blastocyst, Grade IV | 0 (0, 0) | 0 (0, 0) | 0 (0, 0) | 0.52 | 0 (0, 0.003) | 0.771 | 0.826 | -0.011 | 0.661 |

Note: Data are presented as median (interquartile range, IQR). Group comparisons performed using Kruskal–Wallis H test.

† P values adjusted using the Benjamini-Hochberg false discovery rate (FDR) procedure within each variable.

P for trend calculated using Spearman rank correlation test.

IQR: interquartile range; CI: confidence interval.
