# Supplementary material for: Association between laser-assisted hatching and subsequent blastocyst development in fresh day 3 cleavage-stage embryos: a retrospective cohort study using propensity score matching, generalized estimating equations, and time-sensitivity analyses
Source: Front Endocrinol (Lausanne). 2026 Jul 8;17:1871377. doi: 10.3389/fendo.2026.1871377 (PMC13388058; doi:10.3389/fendo.2026.1871377)
Supplement: Supplementary file 5 [file Table5.docx]

**Table S5.** Subgroup analysis of blastocyst development outcomes by infertility type.

| Outcome measure | Secondary (n=985) Median (IQR) | Primary (n=1013) Median (IQR) | Z | \|r\| (95% CI) | P value | Adjusted P value† |
| --- | --- | --- | --- | --- | --- | --- |
| Blastocyst formation, all stages | 0.5 (0.286, 0.667) | 0.5 (0.308, 0.714) | -1.638 | 0.037 (-0.08, 0.007) | 0.101 | 0.253 |
| Transferable blastocyst, all stages | 0.333 (0.125, 0.538) | 0.333 (0.143, 0.571) | -1.154 | 0.026 (-0.07, 0.018) | 0.249 | 0.414 |
| High-quality blastocyst, all stages | 0.125 (0, 0.333) | 0.167 (0, 0.333) | -2.619 | 0.059 (-0.102, -0.015) | 0.009 | 0.056 |
| Blastocyst formation, Grade I | 1 (0.667, 1) | 1 (0.667, 1) | 0.051 | 0.002 (-0.085, 0.09) | 0.960 | 0.960 |
| Transferable blastocyst, Grade I | 1 (0.5, 1) | 1 (0.5, 1) | 0.155 | 0.007 (-0.081, 0.095) | 0.877 | 0.940 |
| High-quality blastocyst, Grade I | 0.5 (0, 1) | 0.5 (0, 1) | -1.067 | 0.048 (-0.135, 0.04) | 0.286 | 0.429 |
| Blastocyst formation, Grade II | 0.75 (0.5, 1) | 0.778 (0.5, 1) | -1.422 | 0.039 (-0.092, 0.015) | 0.155 | 0.332 |
| Transferable blastocyst, Grade II | 0.5 (0.25, 1) | 0.6 (0.333, 1) | -0.655 | 0.018 (-0.071, 0.036) | 0.512 | 0.591 |
| High-quality blastocyst, Grade II | 0.25 (0, 0.5) | 0.3 (0, 0.563) | -1.289 | 0.035 (-0.088, 0.018) | 0.198 | 0.370 |
| Blastocyst formation, Grade III | 0.5 (0, 1) | 0.667 (0, 1) | -2.51 | 0.072 (-0.128, -0.016) | 0.012 | 0.056 |
| Transferable blastocyst, Grade III | 0.333 (0, 0.75) | 0.5 (0, 1) | -2.436 | 0.07 (-0.126, -0.014) | 0.015 | 0.056 |
| High-quality blastocyst, Grade III | 0 (0, 0.333) | 0 (0, 0.5) | -3.31 | 0.095 (-0.151, -0.039) | <0.001 | 0.014 |
| Blastocyst formation, Grade IV | 0.167 (0, 0.5) | 0.25 (0, 0.5) | -1.751 | 0.044 (-0.093, 0.005) | 0.080 | 0.240 |
| Transferable blastocyst, Grade IV | 0 (0, 0.25) | 0 (0, 0.25) | -0.934 | 0.023 (-0.073, 0.026) | 0.351 | 0.440 |
| High-quality blastocyst, Grade IV | 0 (0, 0) | 0 (0, 0) | -0.931 | 0.023 (-0.073, 0.026) | 0.352 | 0.440 |

Note: Data are presented as median (interquartile range, IQR). Group comparisons performed using Wilcoxon rank-sum test (Mann–Whitney U test).

† P values adjusted using the Benjamini-Hochberg false discovery rate (FDR) procedure within each variable.

IQR: interquartile range; CI: confidence interval.
